# Genetic variants in *CETP* increase risk of intracerebral hemorrhage

**DOI:** 10.1002/ana.24780

**Published:** 2016-10-19

**Authors:** Christopher D. Anderson, Guido J. Falcone, Chia‐Ling Phuah, Farid Radmanesh, H. Bart Brouwers, Thomas W. K. Battey, Alessandro Biffi, Gina M. Peloso, Dajiang J. Liu, Alison M. Ayres, Joshua N. Goldstein, Anand Viswanathan, Steven M. Greenberg, Magdy Selim, James F. Meschia, Devin L. Brown, Bradford B. Worrall, Scott L. Silliman, David L. Tirschwell, Matthew L. Flaherty, Peter Kraft, Jeremiasz M. Jagiella, Helena Schmidt, Björn M. Hansen, Jordi Jimenez‐Conde, Eva Giralt‐Steinhauer, Roberto Elosua, Elisa Cuadrado‐Godia, Carolina Soriano, Koen M. van Nieuwenhuizen, Catharina J. M. Klijn, Kristiina Rannikmae, Neshika Samarasekera, Rustam Al‐Shahi Salman, Catherine L. Sudlow, Ian J. Deary, Andrea Morotti, Alessandro Pezzini, Joanna Pera, Andrzej Urbanik, Alexander Pichler, Christian Enzinger, Bo Norrving, Joan Montaner, Israel Fernandez‐Cadenas, Pilar Delgado, Jaume Roquer, Arne Lindgren, Agnieszka Slowik, Reinhold Schmidt, Chelsea S. Kidwell, Steven J. Kittner, Salina P. Waddy, Carl D. Langefeld, Goncalo Abecasis, Cristen J. Willer, Sekar Kathiresan, Daniel Woo, Jonathan Rosand, on behalf of the Global Lipids Genetics Consortium and International Stroke Genetics Consortium

**Affiliations:** ^1^ Center for Human Genetic Research, Massachusetts General Hospital (MGH)BostonMA; ^2^ J. Philip Kistler Stroke Research Center, Department of Neurology, MGHBostonMA; ^3^ Division of Neurocritical Care and Emergency Neurology, Department of Neurology, MGHBostonMA; ^4^ Program in Medical and Population Genetics, Broad InstituteCambridgeMA; ^5^ Departments of Epidemiology and Biostatistics, Harvard T. H. Chan School of Public HealthBostonMA; ^6^ Division of Behavioral Neurology, Department of Neurology, MGHBostonMA; ^7^ Division of Psychiatry, Department of Psychiatry, MGHBostonMA; ^8^ Department of Public Health SciencesInstitute of Personalized Medicine, Penn State College of MedicineHersheyPA; ^9^ Department of Emergency Medicine, MGHBostonMA; ^10^ Department of NeurologyBeth Israel Deaconess Medical CenterBostonMA; ^11^ Department of NeurologyMayo ClinicJacksonvilleFL; ^12^ Stroke Program, Department of NeurologyUniversity of Michigan Health SystemAnn ArborMI; ^13^ Departments of Neurology and Public Health SciencesUniversity of Virginia Health SystemCharlottesvilleVA; ^14^ Department of NeurologyUniversity of Florida College of MedicineJacksonvilleFL; ^15^ Stroke Center, Harborview Medical CenterUniversity of WashingtonSeattleWA; ^16^ Department of NeurologyUniversity of Cincinnati College of MedicineCincinnatiOH; ^17^ Department of NeurologyJagiellonian University Medical CollegeKrakowPoland; ^18^ Institute of Molecular Biology and BiochemistryMedical University GrazGrazAustria; ^19^ Division of Neurology, Department of Clinical Sciences LundLund UniversityLundSweden; ^20^ Division of Neurology, Department of Neurology and Rehabilitation MedicineSkåne University HospitalLundSweden; ^21^ Neurovascular Research Unit, Department of Neurology, Municipal Institute of Medical Investigation–Hospital of the SeaAutonomous University of BarcelonaBarcelonaSpain; ^22^ Program in Inflammation and Cardiovascular Disorders, Municipal Institute of Medical Investigation–Hospital of the SeaAutonomous University of BarcelonaBarcelonaSpain; ^23^ Department of Neurology and Neurosurgery, Brain Center Rudolf MagnusUniversity Medical Center UtrechtUtrechtthe Netherlands; ^24^ Department of NeurologyDonders Institute for Brain, Cognition, and Behavior, Radboud University Medical CenterNijmegenthe Netherlands; ^25^ Division of Clinical Brain SciencesUniversity of EdinburghEdinburghUnited Kingdom; ^26^ Institute for Genetics and Molecular MedicineUniversity of EdinburghEdinburghUnited Kingdom; ^27^ Centre for Cognitive Ageing and Cognitive EpidemiologyUniversity of EdinburghEdinburghUnited Kingdom; ^28^ Department of Clinical and Experimental SciencesNeurology Clinic, University of BresciaBresciaItaly; ^29^ Department of NeurologyMedical University of GrazGrazAustria; ^30^ Division of Neuroradiology, Department of RadiologyMedical University of GrazGrazAustria; ^31^ Neurovascular Research Laboratory and Neurovascular Unit, Research Institute, Vall d'Hebron HospitalAutonomous University of BarcelonaBarcelonaSpain; ^32^ Stroke Pharmacogenomics and Genetics, Terrassa Mutual Teaching and Research Foundation, Terrassa Mutual HospitalTerrassaSpain; ^33^ Department of NeurologyUniversity of ArizonaTucsonAZ; ^34^ Department of NeurologyBaltimore Veterans Administration Medical Center and University of Maryland School of MedicineBaltimoreMD; ^35^ National Institute of Neurological Disorders and StrokeNational Institutes of HealthBethesdaMD; ^36^ Center for Public Health Genomics and Department of Biostatistical SciencesWake Forest UniversityWinston‐SalemNC; ^37^ Center for Statistical Genetics, Department of BiostatisticsUniversity of Michigan School of Public HealthAnn ArborMI; ^38^ Division of Cardiology, Department of Internal MedicineUniversity of Michigan Medical SchoolAnn ArborMI; ^39^ Department of Human GeneticsUniversity of Michigan Medical SchoolAnn ArborMI; ^40^ Cardiovascular Disease Prevention Center, MGHBostonMA

## Abstract

**Objective:**

In observational epidemiologic studies, higher plasma high‐density lipoprotein cholesterol (HDL‐C) has been associated with increased risk of intracerebral hemorrhage (ICH). DNA sequence variants that decrease cholesteryl ester transfer protein (*CETP*) gene activity increase plasma HDL‐C; as such, medicines that inhibit CETP and raise HDL‐C are in clinical development. Here, we test the hypothesis that *CETP* DNA sequence variants associated with higher HDL‐C also increase risk for ICH.

**Methods:**

We performed 2 candidate‐gene analyses of *CETP*. First, we tested individual *CETP* variants in a discovery cohort of 1,149 ICH cases and 1,238 controls from 3 studies, followed by replication in 1,625 cases and 1,845 controls from 5 studies. Second, we constructed a genetic risk score comprised of 7 independent variants at the *CETP* locus and tested this score for association with HDL‐C as well as ICH risk.

**Results:**

Twelve variants within *CETP* demonstrated nominal association with ICH, with the strongest association at the rs173539 locus (odds ratio [OR] = 1.25, standard error [SE] = 0.06, *p* = 6.0 × 10^−4^) with no heterogeneity across studies (*I* ^2^ = 0%). This association was replicated in patients of European ancestry (*p* = 0.03). A genetic score of *CETP* variants found to increase HDL‐C by ∼2.85mg/dl in the Global Lipids Genetics Consortium was strongly associated with ICH risk (OR = 1.86, SE = 0.13, *p* = 1.39 × 10^−6^).

**Interpretation:**

Genetic variants in *CETP* associated with increased HDL‐C raise the risk of ICH. Given ongoing therapeutic development in *CETP* inhibition and other HDL‐raising strategies, further exploration of potential adverse cerebrovascular outcomes may be warranted. Ann Neurol 2016;80:730–740

Serum levels of high‐density lipoprotein cholesterol (HDL‐C) are strongly and inversely associated with coronary artery disease (CAD) risk.^1^ Of the many single nucleotide polymorphisms (SNPs) associated with HDL‐C levels, those within cholesteryl ester transfer protein (*CETP*) have the strongest effect.^2^, ^3^, ^4^ Inhibitory variants within *CETP* associated with increased HDL‐C correlate with reduced risk of multiple cardiac risk factors, including metabolic syndrome and myocardial infarction.^5^, ^6^, ^7^, ^8^ Inhibitors of the CETP gene product, designed to raise HDL‐C by limiting CETP‐mediated exchange of cholesteryl esters and triglycerides between HDL and low‐density lipoprotein (LDL)/very low‐density lipoprotein particles, are being investigated in ongoing phase III trials as treatments to reduce CAD risk.^9^, ^10^

In contrast, substantial data suggest that elevations in HDL‐C may increase risk of spontaneous intracerebral hemorrhage (ICH).^11^, ^12^ Furthermore, clinical trial data suggest an increased risk of ICH on statins despite a lack of significant differences in lipid levels.^13^, ^14^ Because of small sample sizes and confounding by environmental or medical exposures, prior studies have not been able to resolve this potentially paradoxical role of elevated HDL‐C in ICH. Although ICH comprises only 15 to 20% of all strokes, it accounts for 50% of all stroke‐related mortality and 30% of total costs.^15^, ^16^ Blood pressure control remains the only available preventive strategy.^17^ As HDL‐C evolves as a cardiovascular treatment target and clinical trial data on therapeutic modifiers accrue, an improved mechanistic understanding of the pathways involved in hemorrhagic cerebrovascular disease could lead to alternative treatments and prevention strategies for ICH.

It is not known whether CETP inhibitors, which endeavor to produce a biological effect similar to known genetic variants in *CETP*, increase ICH risk. The objective of this study was to use genome‐wide genotypes from individuals with and without ICH from the International Stroke Genetics Consortium (ISGC) to test genetic variants within *CETP* for association with ICH risk, under the hypothesis that the HDL‐raising effects of inhibitory variants within *CETP* will result in increased ICH. *CETP* genetic variants that impact HDL‐C are unconfounded by other exposures, remain constant throughout life, and may be more reflective of long‐term levels than periodic lipid measurements.^18^ Thus, examination of *CETP* genetic variation constitutes a valuable causal inference tool to help strengthen or disclaim prior observations of association between elevated HDL‐C and ICH, and could provide additional clues about potential adverse effects of pharmacologic CETP inhibition.

## Materials and Methods

### Study Design

We performed a 2‐stage (discovery and replication) case–control candidate‐gene association study using both genome‐wide data and direct genotyping. The discovery phase utilized data from 3 genome‐wide association studies (GWASs) of ICH, sampling patients of European ancestry (Table 1).^19^ Replication involved direct genotyping of variants of interest from individuals recruited through 5 case–control studies of ICH, with no overlap between individuals from the discovery phase (Table 2). Detailed description of discovery and replication case and control recruitment architectures can be found in Supplementary Table 1.

**Table 1:** Discovery Populations

	GOCHA		ISGC ICH Study		GERFHS	
Variable	Cases	Controls	Cases	Controls	Cases	Controls
No.	371	389	404	530	374	319
Age, mean (SD)	74 (10)	72 (8)	70 (13)	66 (16)	67 (15)	67 (14)
Female, No. [%]	172 [46]	195 [50]	189 [47]	266 [50]	194 [52]	172 [54]
HTN, No. [%]	274 [75]	227 [58]	278 [69]	247 [47]	241 [64]	166 [52]
T2D, No. [%]	68 [18]	35 [9]	89 [22]	68 [13]	72 [19]	42 [13]
HL, No. [%]	144 [39]	195 [50]	87 [22]	48 [9]	131 [35]	133 [42]
Smoking, No. [%]	56 [15]	15 [4]	58 [14]	74 [14]	79 [21]	46 [14]
Genotyping platform	Illumina 610	Illumina 610	Illumina 610	Illumina 610	Affymetrix 6.0	Affymetrix 6.0
Lobar,^a^ No. [%]	205 [55]	–	135 [33]	–	156 [42]	–

Discovery totals: 2,387 individuals (1,149 cases, 1,238 controls), 43% lobar ICH.

a Lobar ICH location.

GERFHS = Genetic and Environmental Risk Factors for Hemorrhagic Stroke study; GOCHA = Genes and Outcomes of Cerebral Hemorrhage on Anticoagulation study; HL = hyperlipidemia; HTN = hypertension; ICH = intracerebral hemorrhage; ISGC = International Stroke Genetics Consortium; SD = standard deviation; T2D = type 2 diabetes mellitus.

**Table 2:** Replication Populations

	MGH		ERICH		University of Brescia		UMC Utrecht		University of Edinburgh	
Variable	Cases	Controls	Cases	Controls	Cases	Controls	Cases	Controls	Cases	Controls
No.	240	458	920	826	198	185	157	160	110	216
Age, No. (SD)	76 (10)	69 (11)	69 (14)	68 (13)	69 (13)	63 (14)	62 (13)	56 (11)	75 (9)	76 (10)
Female, No. [%]	96 [40]	206 [45]	397 [43]	371 [45]	81 [41]	85 [46]	66 [42]	67 [42]	59 [54]	118 [54]
Lobar, No. [%]	120 [48]	–	380 [41]	–	82 [41]	–	60 [38]	–	61 [55]	–
Genotyping platform	iPLEX	iPLEX	Taqman	Taqman	iPLEX	iPLEX	iPLEX	iPLEX	iPLEX	iPLEX

Replication totals: 3,470 individuals (1,625 cases, 1,845 controls), 42% lobar ICH. Discovery + replication totals: 5,625 individuals (2,595 cases, 3,030 controls), 45% lobar ICH.

ERICH = Ethnic/Racial Variations of Intracerebral Hemorrhage; ICH = intracerebral hemorrhage; iPLEX = Sequenom MassARRAY iPLEX Platform; MGH = Massachusetts General Hospital; SD = standard deviation; TaqMan = Applied Biosystems Taqman Genotyping Assay; UMC = University Medical Center.

All studies had approval from the local institutional review board or ethics committee at each participating institution. Informed consent was obtained from all patients or their legally authorized representatives, or was waived via protocol‐specific allowance.

### Cases

ICH was defined as a new and acute neurological deficit with compatible brain imaging. Enrolled patients were adult consenting primary acute ICH cases that presented to participating institutions with confirmation of primary ICH through computed tomography or magnetic resonance imaging. Exclusion criteria included trauma, brain tumor, hemorrhagic transformation of a cerebral infarction, vascular malformation, or any other cause of secondary ICH in all participating studies.

#### Case Populations

ICH cases were recruited across multiple centers participating in the ISGC from sites in the USA and Europe. For the purposes of reducing confounding by population stratification, only individuals of self‐reported European (Caucasian) ancestry were included in the analysis. Likewise, several studies (Genetics of Cerebral Hemorrhage with Anticoagulation, Edinburgh Stroke Study, LINCHPIN) recruited ICH patients with ICH in the presence of anticoagulation (typically warfarin) exposure. These individuals were excluded from analyses due to the etiopathological distinctness of warfarin‐related primary ICH from other forms. Discovery case populations were enrolled according to methods previously described.^19^ Replication cases were recruited from ISGC participating centers using similar criteria as discovery cases (Supplementary Table 2). Briefly, the University Medical Center (UMC) Utrecht ICH study included additional screening for secondary ICH cases in follow‐up. The Edinburgh Stroke Study recruited subjects aged >55 years only, and specifically excluded individuals with antecedent illicit drug use or presentation >1 week from onset of symptoms. The LINCHPIN study identified ICH cases aged >16 years with acute or chronic ICH from a prospective cohort of individuals living in the Lothian region of Scotland, United Kingdom.

#### Neuroimaging

Stroke neurologists and neuroradiologists at each participating site performed the neuroimaging assessment. Following known differences in underlying biology, ICH was classified as lobar or nonlobar according to location.^20^ ICH originating in the corticosubcortical junction (with or without involvement of subcortical white matter) was defined as lobar, whereas ICH selectively involving the thalamus, internal capsule, basal ganglia, brainstem, or cerebellum was defined as nonlobar.

### Controls

Controls were ICH‐free individuals >18 years of age and were enrolled from the same populations that gave rise to the cases. Controls were confirmed to have no history of previous ICH by interview and/or medical record review. Control population age restrictions were identical to case population age restrictions for all included studies.

#### Control Populations

ICH‐free controls were recruited from the same populations that gave rise to the ICH cases, through inpatient recruitment, ambulatory centers in the local communities, blood donation centers serving the same population, and in the case of the Lothian Birth Cohort, a population cohort study (Supplementary Table 3). The Genetic and Environmental Risk Factors for Hemorrhagic Stroke (GERFHS) and Ethic/Racial Variations of Intracerebral Hemorrhage (ERICH) studies^19^, ^21^ used random digit dialing, the Lothian Birth Cohort individuals were matched to case samples by local investigators,^22^ and UMC Utrecht identified controls from the local blood donor population. The remainder of the studies used random selection from ambulatory clinics or geographically matched populations where cases were being recruited.

### Exposure: Common Genetic Variants within *CETP*

In the discovery phase, we ascertained variants within *CETP* by means of genome‐wide genotyping followed by imputation using methods and quality control procedures previously described.^19^ Briefly, DNA was isolated from fresh or frozen peripheral whole blood collected from cases and controls at each participating institution at the time of consent, quantified with a quantification kit (Qiagen, Valencia, CA), and normalized to a concentration of 30ng/μl. Cases and controls were plated together and genotyped on Illumina (San Diego, CA) 610 or Affymetrix (Santa Clara, CA) 6.0 platforms. Standard quality controls for genome‐wide data were applied, and the resulting set of individuals and SNPs were carried forward to imputation, which was completed using IMPUTE2 with 1000 Genomes–based reference panels (March 2012 version).^23^ Postimputation exclusion filters were minor allele frequency (MAF) < 0.01 and information score < 0.5. SNPs were extracted from the *CETP* gene region according to the human genome reference GRCh38.p2 annotated location (http://www.ncbi.nlm.nih.gov), ± 50 kilobases.

### Independent Replication

*CETP* variants exceeding Bonferroni‐corrected significance and without significant heterogeneity (*I* ^2^ < 40%) for association with ICH in the discovery phase were selected for replication.^24^ Replication SNPs were chosen based on proxy status with index SNPs. Because replication of CETP variants was carried out as part of an ongoing GWAS of ICH, a constraint for the selection of replication SNPs was predicted genotyping success using iPLEX (Sequenom, San Diego, CA) and Taqman (Applied Biosystems, Foster City, CA) methodologies, which were employed at the Massachusetts General Hospital and University of Miami genotyping centers, respectively (see Table 2). Ancestry‐informative markers were also genotyped to facilitate adjustment for population admixture.

### Data Analysis

We present discrete variables as counts (percentage) and continuous variables as mean (standard deviation [SD]) or median (interquartile range), as appropriate.

#### Population Structure

Principal component analysis was implemented in both discovery and replication to account for population structure, using genome‐wide data in discovery and prespecified ancestry‐informative markers in replication.^25^, ^26^ Caucasian population outliers were identified and removed by visual inspection of plots generated with principal components 1 and 2, and these principal components were included as covariates in regression models fitted for association testing. In the GERFHS and ERICH samples, further refinement of population structure was achieved using the ADMIXTURE software tool to remove outliers.^27^

#### Association Testing

Prior to discovery association testing, SNPs within *CETP* were clumped into loci sharing linkage disequilibrium (LD) *r* ^2^ > 0.5 using PLINK to allow discrimination of semi‐independent loci across the gene. Association testing for SNPs within the *CETP* locus and ICH risk was completed separately for all ICH, as well as for lobar and nonlobar hemorrhages. Logistic regression models were fitted assuming independent additive genetic effects for dosage of the minor allele (1 degree of freedom additive trend test), and adjusted for age, gender, and principal components 1 and 2. A similar analytic approach was undertaken for analysis of replication data, using additive allele genotype data rather than dosage.

#### Meta‐Analysis

Fixed effects, inverse variance weighted meta‐analysis was used to pool effect estimates across studies, assessing heterogeneity by computing Cochrane's *Q* (with corresponding *p*) and *I* ^2^ (percentage of effect size attributable to heterogeneity). Identical meta‐analysis procedures were used for pooling of effects across studies in discovery and replication, and across all studies.^28^

### Genetic Risk Score Analysis

Variants within the *CETP* locus with established association with HDL‐C levels in the most recent Global Lipids Genetics Consortium (GLGC) analysis (unpublished data) were extracted from the discovery data set and tested for association with ICH using an additive multi‐SNP genetic risk score approach using the GTX package (http://CRAN.R-project.org/package=gtx) in R (version 3.0). Ten variants surpassing exome array‐wide significance (*p* < 2.1 × 10^−7^) and demonstrating independence using a sequential forward selection model in the GLGC data set were identified, of which 7 were available in our ICH discovery data set.^29^ These 7 variants, on average, were associated with a 0.19‐SD increase in HDL‐C (∼2.85mg/dl) in the GLGC population (*p* < 1 × 10^−200^). This corresponds to a proportion of variance explained of 0.032. ICH risk was predicted from summary statistics, weighted according to the established HDL‐C effect, and oriented to the HDL‐C increasing allele.

### Statistical Testing and Software

We used a conservative Bonferroni‐corrected threshold for statistical significance of *p* < 0.004, adjusted for the number of semi‐independent loci within *CETP* with *r* ^2^ < 0.5 (12 tests in this analysis). Quality control procedures, genetic association testing for single variants, and score calculations were performed in SNPTest and PLINK v1.07.^26^, ^30^ Imputation was completed using IMPUTE2.^23^ All other statistical analyses were performed in SAS 9.2 (SAS Institute, Cary, NC).

## Results

Following relevant exclusions during quality control and principal component analysis, 1,149 ICH cases and 1,238 controls from 3 case–control studies of ICH were included in the discovery phase, 43% of which were of the lobar ICH subtype (see Table 1).

### *CETP* Genetic Variants

After imputation using 1000 Genomes reference panels and application of genome‐wide quality control filters, a total of 390 common variants of MAF > 0.01 were extracted from the *CETP* gene and 50kb flanking regions (Supplementary Table 4).^31^ These 390 variants were present either via array‐based ascertainment or imputation in all 3 of the discovery data sets, and were used for association testing.

### Single‐SNP Association Testing

**Figure 1:**
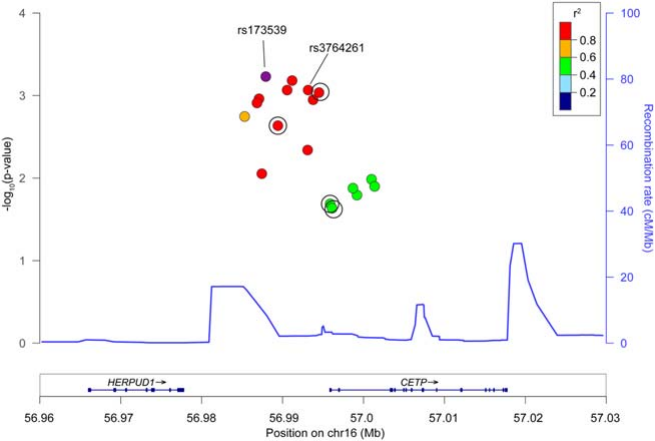
Regional association plot of rs173539 and single nucleotide polymorphisms (SNPs) exhibiting *r* ^2^ > 0.5 in association with intracerebral hemorrhage. SNPs available for replication are circled. Mean recombination rate across the locus is represented by the continuous line. The rs3764261 variant identified was the leading SNP in prior genome‐wide association studies of high‐density lipoprotein cholesterol. chr = chromosome; cM = centimorgans; Mb = megabase. [Color figure can be viewed in the online issue, which is available at www.annalsofneurology.org.]

After testing all 390 SNPs within *CETP* clumped into regions sharing *r* ^2^ > 0.5, 12 loci demonstrating nominal association with ICH (*p* < 0.05) were identified (Supplementary Table 5). Three of these loci surpassed Bonferroni correction (Table 3) with residual *r* ^2^ = 0.25 to 0.39 between them. Among these, only rs173539 (odds ratio [OR] = 1.25, standard error [SE] = 0.06, *p* = 6.00 × 10^−4^) met prespecified criteria for replication due to its homogeneity across discovery data sets (*I* ^2^ = 0%). Of note, rs173539 was in high LD with rs3764261 (*r* ^2^ = 0.98), the strongest associated SNP with HDL‐C in published GWASs of lipid levels (Fig 1).^32^ Comparison of effects of the rs173539 locus on risk of lobar versus nonlobar hemorrhage revealed no significant differences by ICH subtype (Supplementary Table 6).

**Table 3:** Discovery *CETP* Loci Demonstrating Bonferroni‐Significant Association with ICH

Lead SNP	CHR	Tested Allele	MAF	Effect Direction	OR	SE	Discovery *p*	*I* ^2^
rs173539	16	T	0.31	+++	1.25	0.06	6.00E‐4	0
rs820299	16	G	0.38	−−−	0.81	0.06	7.50E‐4	48
rs158478	16	A	0.48	+++	1.21	0.06	1.48E‐3	56

+ = variant increases ICH risk; − = variant decreases ICH risk; CHR = chromosome; ICH = intracerebral hemorrhage; MAF = minor allele frequency; OR = odds ratio; SE = standard error; SNP = single nucleotide polymorphism.

### Replication and Meta‐Analysis of the rs173539 Locus

A total of 1,625 ICH cases and 1,845 controls of Caucasian ancestry were available for replication. Following application of predictive algorithms for SNP genotype ascertainment success using both genotyping methodologies employed, 4 SNPs in LD with rs173539 locus were selected for replication genotyping according to the constraints described (Tables 4 and 5). Both rs173539 and rs3764261 were predicted to fail in one or both replication pools. All 4 selected SNPs were successfully genotyped in all replication data sets. All replication results showed minimal heterogeneity and consistent directions of effect, and 2 variants replicated at *p* < 0.05. In meta‐analysis, all 4 SNPs within the rs173539 locus chosen for replication were strengthened by addition of the replication SNP data, with minimal heterogeneity in the final total sample size of 2,595 ICH cases and 3,030 controls (see Table 5).

**Table 4:** Discovery SNP rs173539 and Local Proxies in Association with ICH Risk

SNP	CHR	Tested Allele	MAF	Effect Direction	OR	SE	Discovery *p*	*I* ^2^
rs173539	16	T	0.31	+++	1.25	0.06	6.00 × 10^−4^	0
−rs247617, *r* ^2^ = 0.99	16	A	0.31	+++	1.24	0.06	8.74 × 10^−4^	0
−rs17231506, *r* ^2^ = 0.99	16	T	0.31	+++	1.23	0.06	9.13 × 10^−4^	0
−rs711752, *r* ^2^ = 0.62	16	A	0.42	++−	1.15	0.06	2.08 × 10^−2^	14
−rs708272, *r* ^2^ = 0.61	16	A	0.42	++−	1.15	0.06	2.23 × 10^−2^	18

Association results for rs173539 in association with ICH risk, as well as 4 additional SNPs in LD with rs173539 chosen for replication.

− = variant decreases ICH risk; + = variant increases ICH risk; CHR = chromosome; ICH = intracerebral hemorrhage; LD = linkage disequilibrium; MAF = minor allele frequency; OR = odds ratio; *r* ^2^ = degree of LD with rs173539; SE = standard error; SNP = single nucleotide polymorphism.

**Table 5:** Replication Results for SNPs in Linkage Disequilibrium with rs173539 and Meta‐Analysis of All Samples

Replication						Discovery/Replication Meta‐Analysis				
SNP	Effect	OR	SE	*p*	*I* ^2^	Effect	OR	SE	*p*	*I* ^2^
rs247617	+++++	1.08	0.05	0.18	2	+++/+++++	1.13	0.04	1.0 × 10^−3^	0
rs17231506	+++++	1.08	0.05	0.17	1	+++/+++++	1.13	0.04	1.0 × 10^−3^	0
rs711752	++++−	1.12	0.05	0.03	7	++−/++++−	1.13	0.04	1.0 × 10^−3^	0
rs708272	++++−	1.14	0.05	0.01	4	++−/++++−	1.14	0.04	5.0 × 10^−4^	0

+ = variant increases ICH risk; − = variant decreases ICH risk; ICH = intracerebral hemorrhage; OR = odds ratio; SE = standard error; SNP = single nucleotide polymorphism.

### Genetic Risk Score Analysis

An additive multi‐SNP genetic risk score was constructed using independent HDL‐association data.^29^ Ten variants were selected, of which 7 were present in the ICH discovery data set (Table 6). Three variants were unavailable in the ICH data set due to differences in genotyping platforms (exome array vs GWAS array) between the two studies. The genetic risk score of these 7 variants demonstrated association with ICH (OR = 1.86, SE = 0.13, *p* = 1.39 × 10^−6^).

**Table 6:** ICH Association Results for Variants of Known HDL‐C Effect Used to Compute Genetic Risk Score

SNP	Ref Allele	MAF	ICH OR	ICH Beta	ICH SE	ICH *p*	HDL Effect Allele	HDL Beta	HDL SE	Type
rs173539	T	0.31	1.25	0.222	0.065	0.0006	T	0.230	0.0028	Intergenic
rs3764261	A	0.31	1.23	0.210	0.063	0.0009	A	0.239	0.0028	Intergenic
rs247616	T	0.30	1.22	0.196	0.064	0.0023	T	0.242	0.0028	Intergenic
rs9989419	A	0.40	0.92	−0.079	0.059	0.1808	G	0.131	0.0026	Intergenic
rs5880	C	0.04	1.22	0.202	0.151	0.1812	G	0.258	0.0067	Nonsyn.
rs5882	G	0.32	1.06	0.057	0.065	0.3803	G	0.092	0.0028	Nonsyn.
rs7499892	T	0.19	1.02	0.022	0.076	0.7758	C	0.230	0.0033	Intronic

HDL‐C = high‐density lipoprotein cholesterol; ICH = intracerebral hemorrhage; MAF = minor allele frequency; Nonsyn. = nonsynonymous; OR = odds ratio; Ref = reference; SE = standard error; SNP = single nucleotide polymorphism.

## Discussion

Our results demonstrate an association between *CETP* gene variants in the rs173539 locus and risk of ICH, opposite in direction from their effect on risk of CAD and metabolic syndrome.^5^, ^7^, ^8^ Furthermore, an aggregated score of variants within *CETP* that raise HDL‐C is strongly associated with increased ICH risk. These results suggest that there may be substantial differences in the roles of lipids in the progression of cerebrovascular and cardiometabolic diseases. Novel therapies targeting CETP along with other approaches to increase HDL‐C are currently under active investigation in an effort to reduce the risk of CAD.^33^ Because the cerebral small vessel diseases that lead to ICH are common in the aging population and frequently coincide with risk factors for cardiometabolic disease,^34^, ^35^ our observations supporting opposing effects of HDL‐C on ICH and CAD underscore the need for a better understanding of which patients could be at increased risk of ICH on therapies aimed at increasing HDL‐C.

Our findings support prior studies linking elevated HDL‐C with increased risk of ICH. Unlike prior studies, however, our genetic approach limits confounding by dietary, environmental, or medication exposures. A recent meta‐analysis of epidemiological studies examining associations between cholesterol levels and ICH found a dose–response relationship between HDL‐C and ICH risk, with each 1mmol/l increase in HDL‐C associated with a 17% increase in ICH risk.^11^ This result was nullified when studies of subarachnoid hemorrhage patients were included, but strengthened by restriction to studies from the United States, highlighting the potential confounds of case misspecification and unmeasured environmental exposures in testing associations of this nature.

HDL‐C appears to have a complex and context‐dependent role in cerebrovascular disease. In contrast to ICH, elevated HDL‐C is associated with reduced risk of ischemic stroke, particularly strokes caused by large artery atherosclerotic disease, consistent with the observed associations of HDL‐C in CAD.^36^ However, Mendelian randomization (MR) studies of genetic variants predisposing to elevated HDL‐C have not demonstrated association with either ischemic stroke or CAD, suggesting the observed relationships may not be causal.^37^, ^38^ Unfortunately, the limited sample size of genetics efforts in ICH coupled with acute changes in lipid values around the time of ICH currently preclude the use of this MR approach in our analyses.^39^

No study, including the present, has yet established a direct causal relationship between HDL‐C and ICH risk. Although associations between *CETP* genetic variants and ICH are almost certainly unidirectional due to the immutability of the genetic code, they still could impact an unseen risk factor that lies outside of the known HDL‐C level determining effects of the gene. Even if causality can be ultimately established, the mechanism by which a CETP‐mediated increase in HDL‐C may worsen ICH risk remains unclear. Inhibition of CETP results in changes to HDL particle size and cholesterol efflux capacity in addition to the observed changes in HDL‐C serum levels, and it may be through these accompanying changes in HDL function that ICH risk is conferred.^40^ Furthermore, accumulating evidence suggests that HDL effects on endothelium are dynamic and modifiable, and can even become proinflammatory with the incorporation of serum amyloid A1, complement C3, and ceramides, resulting in altered immune regulation and reduced antioxidant effects.^41^, ^42^ It is therefore possible that elevated HDL‐C provides a platform to further the vascular inflammatory processes that play a substantial role in the cerebral small vessel disease underlying ICH.^43^ Further studies will be needed to dissect the pathways intersecting with HDL‐C to clarify the foundational biology of its role in ICH.

Therapeutic development of small molecule and biologic compounds designed to raise HDL‐C continue.^44^ Although the first wave of phase III trials of CETP inhibitors were plagued by off‐target effects and futility,^45^ the REVEAL trial of anacetrapib was recently continued after unblinded interim review. Other HDL‐raising strategies, including apolipoprotein‐A1 (ApoA1)‐rich reconstituted HDL particle infusions and ApoA1‐mimetic peptides, continue to be evaluated in preclinical and early phase trials.^44^ Given this pipeline of HDL‐based therapeutic development, it is imperative that potential adverse clinical effects of such strategies be clarified. Early experiences with US Food and Drug Administration–approved PCSK‐9 inhibitors have led to predictions of widespread adoption of this new class of drugs, and it is reasonable to expect that HDL‐C targeted treatments would be no different, resulting in a potentially large population of aging individuals with pharmacologically induced high HDL‐C levels of uncertain long‐term cerebrovascular risk.^46^ The proportion of variance in HDL‐C levels explained by our genetic risk score was 0.032. This is roughly commensurate with observed effects of statins, which in clinical trials raised HDL by 0.04 to 0.10mg/dl.^47^ With emerging HDL‐C modifying strategies likely to exert more profound effects, the impact on ICH risk, if confirmed and verified to be causal, could be more substantial than indicated by our *CETP* genetic risk score.

As noted above, our study cannot determine whether the observed association between *CETP* and ICH risk is through HDL‐C alone. Although they exhibit their largest effect on HDL‐C levels, *CETP* variants are also associated with LDL, triglycerides, and total cholesterol levels.^3^ Although we cannot perform formal MR, the association between our HDL‐C increasing genetic risk score at *CETP* and risk of ICH provides support for an HDL‐specific effect. Even with this suggestion of HDL‐C specificity, the composition of HDL particles can vary with respect to ratios of esterified to unesterified cholesterol as well as apolipoprotein content. Genetic variation that determines circulating HDL‐C does not necessarily capture these secondary characteristics, which could have a substantial impact on biological effects.

An additional limitation of our study is the aggregation of case and control data across multiple sites, which could result in biases between cases and controls. We have attempted to control for study demographics and population structure in our regression analyses, and performed independent replication, but unmeasured confounding could still have impacted the observed associations. Related to this point, all analyses presented were in individuals of European ancestry due to small study populations, and therefore low statistical power, in individuals of other racial and ethnic backgrounds. As a result, our findings cannot be extended to minority populations at this time.

Although our study utilized genome‐wide data for discovery and genetic risk score analyses, our approach was fundamentally a candidate gene study of *CETP*. Using GWAS data allowed for control of population stratification, which can be a major confounder in traditional candidate gene designs employing only direct genotyping. However, it was still based on an a priori hypothesis about *CETP* association with ICH. Therefore, the false discovery rate for association between variants at *CETP* and ICH risk, although stringently controlled using Bonferroni correction at the *CETP* locus, may still be elevated in comparison with a standard GWAS. Due to the hypothesis‐driven nature of our study, we by definition cannot provide novel results about lipid‐related genetic loci that lie outside of the tested gene region.

Finally, the *CETP* gene contains several independent loci which have been associated with lipid levels and clinical endpoints.^3^, ^5^, ^7^, ^32^ This resulted in a more complex replication phase than would have been needed if the genetic architecture of the locus were centered about a single region of association. Coupled with the limitations of variant selection in our replication phase, we cannot distinguish a culprit variant to the exclusion of others. Although all variants chosen for replication demonstrated refined effect size estimates and greater statistical significance in meta‐analysis with discovery data, replication was strongest for variants in slightly lower LD than the lead variant from discovery, and with slightly higher between‐study heterogeneity. Whether this observation represents true heterogeneity of effect at the replicated variants will depend on future validation and extension studies.

We have demonstrated an association between genetic variants in *CETP* and risk of ICH, and have shown that *CETP*'s HDL‐C raising effects could play a role in the pathogenesis of ICH. Further work will be needed to identify how the biological pathways impacted by HDL‐C may impart increased risk of hemorrhage. These pathways may yield crucial novel targets for prevention of ICH and the cerebral small vessel diseases that lead to vessel rupture.

## Author Contributions

C.D.A. and G.J.F. contributed equally to the presented work. Conception and design of study: C.D.A., G.J.F., C.‐L.P., F.R., A.B., G.M.P., S.K., D.W., J. Ros. Acquisition and analysis of data: all authors. Drafting manuscript and figure: C.D.A., G.J.F., C.‐L.P., F.R., A.B., G.M.P., A.M.A., S.K., J.Ros.

## Potential Conflicts of Interest

Nothing to report.

## Supporting information

Additional supporting information can be found in the online version of this article

Supporting Information
