# Supplementary material for: Genetic variants in CETP increase risk of intracerebral hemorrhage
Source: Ann Neurol. 2016 Oct 19;80(5):730–40. doi: 10.1002/ana.24780 (PMC5115931; doi:10.1002/ana.24780)
Supplement: Supplementary file 1 — Supporting Information [file ANA-80-730-s001.docx]

**SUPPLEMENTARY DATA**

**Genetic Variants in *CETP* Increase Risk of Intracerebral Hemorrhage**

CD Anderson et al.

**TABLE OF CONTENTS**

**Supplementary Table S1.** ICH Case and control recruitment architectures for participating studies, and inclusion/exclusion criteria………………………………………………………...**Page 3**

**Supplementary Table S2.** ICH case inclusion and exclusion criteria by site………….…..**Page 4**

**Supplementary Table S3.** Control inclusion and exclusion criteria by site……………...…**Page 5**

**Supplementary Table S4.** Discovery phase association results for all SNPs in *CETP*…..**Page 6**

**Supplementary Table S5.** Discovery phase results for top SNPs within each semi-independent *CETP* locus with ICH risk………………………………………………………………………..**Page 16**

**Supplementary Table S6.** Discovery phase association results for top SNPs within each semi-independent *CETP* locus, stratified by ICH location..........................................................**Page 17**

**Supplementary Table S7.** International Stroke Genetics Consortium Contributors.........**Page 18**

| **Supplementary Table S1. ICH case and control recruitment architectures for participating studies** | | | | |
| --- | --- | --- | --- | --- |
| **Study** | **Participating Centers** | **Population Ages** | **Case Recruitment** | **Control Recruitment** |
| Brescia Stroke Registry | University of Brescia, Brescia, Italy | 18+ | Hospital-based, prospective | Regionally matched, hospital and ambulatory clinics |
| UMC Utrecht ICH Study | University Medical Center Utrecht, Utrecht, The Netherlands | 18+ | Hospital-based, prospective | Regionally matched, blood donor population |
| Edinburgh Stroke Study | Western General Hospital, Edinburgh, Scotland, UK | 55+ | Inpatient and outpatient hospital-based, prospective | N/A |
| LINCHPIN | Western General Hospital, Royal Infirmary of Edinburgh, St. John’s Hospital at Howden, West Lothian, Scotland, UK | 16+ | Community-based in areas served by NHS Lothian Health Board, prospective with hot-pursuit and retrospective augmentation | N/A |
| Lothian Birth Cohort 1936 | All centers serving the Lothian Area of Scotland | 76 years old (cohort assessed at ages 70, 73, and 76) | N/A | Community population born in 1936 who took Scottish Mental Survey in 1947, living in Lothian, Scotland, UK |
| ERICH | 19 centers in USA, based at University of Cincinnati | 18+ | Hospital-based, prospective with hot-pursuit | Regionally matched, random-digit-dialing |
| GOCHA | 6 centers in USA, based at Massachusetts General Hospital | 55+ | Hospital-based, prospective | Regionally matched, ambulatory clinics |
| GERFHS | 16 centers in the Greater Cincinnati/Northern Kentucky region of USA, based at University of Cincinnati | 18+ | Hospital-based, prospective | Regionally matched, random-digit-dialing |
| ISGC Europe | Hospital del Mar ICH study, Vall d’Hebron ICH study in Barcelona, Spain, Jagiellonian University Hemorrhagic Stroke Study in Krakow, Poland, Lund Stroke Register in Lund, Sweden | 18+ | Hospital-based, prospective | Regionally matched, hospital and ambulatory clinics |

| **Supplementary Table S2. ICH case inclusion and exclusion criteria by recruitment site** | | |
| --- | --- | --- |
| **Study** | **Inclusion Criteria** | **Exclusion Criteria** |
| Brescia Stroke Registry | - Acute hospitalization for ICH - CT or MRI confirmation of ICH - Age > 18 | - Head trauma - Brain tumor - Ischemic stroke - Vascular malformation - Other cause of secondary ICH |
| UMC Utrecht ICH Study | - Acute hospitalization for ICH - CT confirmation of ICH - Age > 18 | - Head trauma - Brain tumor - Ischemic stroke - Vascular malformation - Other cause of secondary ICH present on admission or in follow-up |
| Edinburgh - ESS | - Acute hospitalization for ICH - CT or MRI confirmation of ICH - Age > 55 | - Head trauma - Brain tumor - Ischemic stroke - Vascular malformation - Presentation > 1 week from ICH - Antecedent drug use - Primary coagulopathy |
| Edinburgh - LINCHPIN | - Symptomatic ICH (acute or chronic) - CT or MRI confirmation of acute or chronic ICH - Age > 16 - Resident in area served by NHS Lothian Health Board at time of ICH | - Head trauma - Brain tumor - Ischemic stroke with hemorrhagic transformation - Vascular malformation - Other cause of secondary ICH |
| ERICH | - Acute hospitalization for ICH - CT or MRI confirmation of ICH - Age > 18 | - Head trauma - Brain tumor - Ischemic stroke - Vascular malformation - Other cause of secondary ICH |
| GOCHA | - Acute hospitalization for ICH - CT or MRI confirmation of ICH - Age > 55 | - Head trauma - Brain tumor - Ischemic stroke - Vascular malformation - Other cause of secondary ICH |
| GERFHS | - Acute hospitalization for ICH - CT or MRI confirmation of ICH - Age > 18 | - Head trauma - Brain tumor - Ischemic stroke - Vascular malformation - Other cause of secondary ICH |
| ISGC Europe ICH studies (Hospital del Mar, Vall d’Hebron Hospital, Jagiellonian University, Lund University) | - Acute hospitalization for ICH - CT or MRI confirmation of ICH - Age > 18 | - Head trauma - Brain tumor - Ischemic stroke - Vascular malformation - Other cause of secondary ICH |

| **Supplementary Table S3. Control inclusion and exclusion criteria by recruitment site** | | |
| --- | --- | --- |
| **Study** | **Ascertainment Methods** | **Inclusion Criteria** |
| Brescia Stroke Registry | - Screened and collected from the same hospital as ICH cases | - Absence of stroke history, confirmed through interview and review of medical records |
| UMC Utrecht ICH Study | - Blood donors presenting to the same hospital as ICH cases, from same surrounding population | - Healthy blood donor as confirmed through screening questionnaires at the donation facility |
| Edinburgh - Lothian Birth Cohort 1936 | - Individuals in Lothian born in 1936, totaling 1091 participants in Wave 1 at age 70 years, with follow-up waves at ages 73 and 76 | - Random selection matched 2:1 with ICH cases from ESS and LINCHPIN, confirmed stroke-free at age 76 |
| ERICH | - Ascertained through random digit dialing in the regions surrounding centers where cases were recruited, age > 18 | - Absence of ICH history confirmed through interview at the time of consent |
| GOCHA | - Screened and collected from ambulatory clinics at the same centers that recruited cases, age > 55 | - Absence of ICH history confirmed through interview at the time of consent |
| GERFHS | - Ascertained through random digit dialing in the Greater Cincinnati-Northern Kentucky region where cases were recruited, age > 18 | - Absence of ICH history confirmed through interview at the time of consent |
| ISGC Europe ICH studies (Hospital del Mar, Vall d’Hebron Hospital, Jagiellonian University, Lund University) | - Screened and collected from ambulatory clinics at the same centers that recruited cases, age > 18 | - Absence of ICH history confirmed through interview at the time of consent |

| **Supplementary Table S4. Discovery phase association results for all SNPs in *CETP*** | | | | | |
| --- | --- | --- | --- | --- | --- |
| **SNP** | **OR** | **SE** | **P** | **Direction** | **I2** |
| rs173539 | 1.25 | 0.06 | 0.00060 | +++ | 0 |
| rs183130 | 1.24 | 0.06 | 0.00066 | +++ | 0 |
| rs820299 | 0.81 | 0.06 | 0.00075 | --- | 48.1 |
| rs3764261 | 1.23 | 0.06 | 0.00086 | +++ | 0 |
| rs247617 | 1.24 | 0.06 | 0.00087 | +++ | 0 |
| rs17231506 | 1.23 | 0.06 | 0.00091 | +++ | 0 |
| rs821840 | 1.24 | 0.07 | 0.00111 | +++ | 0 |
| rs56156922 | 1.24 | 0.07 | 0.00111 | +++ | 0 |
| rs12446515 | 1.24 | 0.07 | 0.00123 | +++ | 0 |
| rs158478 | 1.21 | 0.06 | 0.00148 | +++ | 56 |
| rs72786786 | 1.24 | 0.07 | 0.00178 | +++ | 0 |
| rs60545348 | 0.82 | 0.07 | 0.00200 | --- | 36.2 |
| rs158479 | 1.20 | 0.06 | 0.00228 | +++ | 14.9 |
| rs247616 | 1.22 | 0.06 | 0.00229 | +++ | 0 |
| rs12597002 | 0.82 | 0.07 | 0.00248 | --- | 36.8 |
| rs708273 | 0.82 | 0.07 | 0.00272 | --- | 11.7 |
| rs4369653 | 0.83 | 0.07 | 0.00390 | --- | 51.7 |
| rs12149545 | 1.20 | 0.06 | 0.00467 | +++ | 0 |
| rs158477 | 1.19 | 0.06 | 0.00542 | +++ | 41 |
| rs56228609 | 1.19 | 0.07 | 0.00904 | +++ | 0 |
| rs4784745 | 0.85 | 0.06 | 0.00973 | --- | 0 |
| rs4784741 | 1.17 | 0.06 | 0.01014 | +++ | 16.6 |
| rs291044 | 0.85 | 0.06 | 0.01180 | --- | 0 |
| rs12444012 | 1.16 | 0.06 | 0.01211 | ++- | 26.5 |
| rs12720926 | 1.16 | 0.06 | 0.01338 | +++ | 0 |
| rs291043 | 0.86 | 0.06 | 0.01340 | --- | 0 |
| rs11508026 | 1.16 | 0.06 | 0.01611 | +++ | 3.8 |
| rs7187261 | 1.46 | 0.16 | 0.01636 | +++ | 0 |
| rs711752 | 1.15 | 0.06 | 0.02079 | ++- | 14 |
| rs708272 | 1.15 | 0.06 | 0.02231 | ++- | 18.5 |
| rs289751 | 1.50 | 0.18 | 0.02310 | ++- | 19.3 |
| rs711751 | 0.87 | 0.06 | 0.02725 | --- | 0 |
| rs4783962 | 0.86 | 0.07 | 0.02749 | --- | 0 |
| rs12447839 | 0.86 | 0.07 | 0.02944 | --- | 0 |
| rs11860407 | 1.14 | 0.06 | 0.03670 | +++ | 0 |
| rs12708980 | 1.13 | 0.06 | 0.03748 | +++ | 0 |
| rs891144 | 1.80 | 0.29 | 0.03975 | +++ | 0 |
| rs4587963 | 0.87 | 0.07 | 0.04051 | --- | 0 |
| rs2033254 | 1.13 | 0.06 | 0.04137 | +++ | 0 |
| rs71387147 | 0.77 | 0.13 | 0.04162 | --- | 0 |
| rs247618 | 0.86 | 0.07 | 0.04195 | --- | 0 |
| rs12447924 | 0.87 | 0.07 | 0.04207 | --- | 0 |
| rs1800775 | 1.12 | 0.06 | 0.04412 | +++ | 0 |
| rs289746 | 1.16 | 0.07 | 0.04637 | +++ | 30.5 |
| rs1800776 | 0.79 | 0.12 | 0.04787 | --- | 14.4 |
| rs7187275 | 1.36 | 0.16 | 0.05059 | +++ | 0 |
| rs12934552 | 0.84 | 0.09 | 0.05069 | --- | 0 |
| rs3816117 | 1.12 | 0.06 | 0.05256 | +++ | 0 |
| rs12708985 | 1.18 | 0.09 | 0.05326 | +++ | 0 |
| rs13337445 | 0.80 | 0.12 | 0.05934 | --+ | 58.5 |
| rs1122390 | 0.87 | 0.07 | 0.05950 | --- | 0 |
| rs289742 | 1.18 | 0.09 | 0.06317 | +++ | 0 |
| rs12447620 | 1.17 | 0.09 | 0.06683 | +++ | 0 |
| rs1800777 | 1.31 | 0.16 | 0.07862 | +++ | 27.7 |
| rs17369163 | 0.81 | 0.12 | 0.07962 | --+ | 41.9 |
| rs1800774 | 1.11 | 0.06 | 0.08460 | +++ | 0 |
| rs4784751 | 1.12 | 0.06 | 0.08496 | ++- | 25.9 |
| rs7197864 | 0.85 | 0.09 | 0.08917 | --- | 0 |
| rs17290922 | 0.85 | 0.09 | 0.08959 | --- | 0 |
| rs4784750 | 1.11 | 0.06 | 0.10300 | ++- | 22.6 |
| rs1651663 | 1.12 | 0.07 | 0.10340 | ++- | 0 |
| rs7205459 | 0.86 | 0.09 | 0.10340 | --- | 0 |
| rs35926917 | 0.82 | 0.12 | 0.10970 | --+ | 30 |
| rs74023630 | 0.86 | 0.10 | 0.11230 | --- | 0 |
| rs9936680 | 0.83 | 0.12 | 0.11520 | --+ | 27 |
| rs158617 | 1.15 | 0.09 | 0.11720 | +++ | 0 |
| rs72786778 | 0.78 | 0.16 | 0.11730 | +-- | 0 |
| rs158480 | 1.15 | 0.09 | 0.12480 | +++ | 0 |
| rs56208677 | 1.21 | 0.12 | 0.12670 | +++ | 0 |
| rs12924030 | 0.87 | 0.09 | 0.12750 | --- | 0 |
| rs77751805 | 1.41 | 0.23 | 0.12930 | +++ | 0 |
| rs12445252 | 1.10 | 0.07 | 0.13250 | ++- | 12.7 |
| rs12444396 | 1.10 | 0.07 | 0.13630 | ++- | 22.1 |
| rs12923459 | 0.91 | 0.06 | 0.13720 | --- | 0 |
| rs9924087 | 0.84 | 0.12 | 0.13980 | --+ | 35 |
| rs289734 | 0.89 | 0.08 | 0.14040 | --- | 0 |
| rs1436425 | 1.10 | 0.07 | 0.14810 | +++ | 0 |
| rs17231534 | 0.80 | 0.15 | 0.15140 | --+ | 31.6 |
| rs74021897 | 1.10 | 0.07 | 0.15200 | +++ | 0 |
| rs289736 | 1.13 | 0.09 | 0.15400 | ++- | 0 |
| rs1684576 | 1.09 | 0.06 | 0.16500 | ++- | 43.4 |
| rs72778371 | 0.89 | 0.08 | 0.16690 | --+ | 0 |
| rs5030708 | 0.77 | 0.19 | 0.17420 | --+ | 0 |
| rs12448528 | 0.90 | 0.08 | 0.17550 | --- | 0 |
| rs11862052 | 1.13 | 0.09 | 0.17610 | +++ | 0 |
| rs9989419 | 0.92 | 0.06 | 0.18080 | +-- | 0 |
| rs5880 | 1.22 | 0.15 | 0.18120 | ++- | 33.3 |
| rs117398617 | 0.90 | 0.08 | 0.18750 | --+ | 5.5 |
| rs891140 | 0.92 | 0.06 | 0.18850 | --+ | 33.2 |
| rs1875236 | 1.16 | 0.11 | 0.19390 | +++ | 0 |
| rs11644475 | 1.33 | 0.22 | 0.19900 | ++- | 44.9 |
| rs289735 | 1.09 | 0.07 | 0.20350 | +-- | 59 |
| rs4471669 | 1.09 | 0.07 | 0.20370 | +++ | 0 |
| rs11644171 | 1.09 | 0.07 | 0.20530 | ++- | 34.7 |
| rs7203984 | 1.10 | 0.08 | 0.20690 | -++ | 61.6 |
| rs289750 | 1.09 | 0.07 | 0.20860 | +-- | 61.5 |
| rs1875235 | 1.15 | 0.11 | 0.21210 | +++ | 0 |
| rs78921879 | 1.13 | 0.10 | 0.22040 | ++- | 0 |
| rs1684575 | 1.08 | 0.06 | 0.22380 | ++- | 78.5 |
| rs289749 | 1.09 | 0.07 | 0.22420 | +-- | 64.5 |
| rs9925054 | 0.93 | 0.06 | 0.22660 | --- | 0 |
| rs1549669 | 0.93 | 0.06 | 0.23250 | --- | 0 |
| rs166017 | 1.08 | 0.07 | 0.25260 | ++- | 32 |
| rs289714 | 1.10 | 0.08 | 0.25690 | +++ | 0 |
| rs7200805 | 0.81 | 0.19 | 0.26620 | --- | 0 |
| rs37025 | 0.93 | 0.06 | 0.27070 | --+ | 0 |
| rs289741 | 1.07 | 0.07 | 0.27560 | +++ | 0 |
| rs28504436 | 1.08 | 0.07 | 0.28780 | ++- | 0 |
| rs1672865 | 1.06 | 0.06 | 0.29450 | ++- | 79.6 |
| rs4783961 | 1.06 | 0.06 | 0.29560 | -++ | 70.3 |
| rs72773107 | 1.17 | 0.15 | 0.30590 | +++ | 0 |
| rs61738710 | 0.87 | 0.13 | 0.30930 | --+ | 0 |
| rs7194225 | 0.88 | 0.12 | 0.31030 | --+ | 43.4 |
| rs9921780 | 1.06 | 0.06 | 0.31310 | +-+ | 46.7 |
| rs172337 | 1.12 | 0.12 | 0.32300 | ++- | 0 |
| rs247610 | 0.94 | 0.06 | 0.32320 | --- | 0 |
| rs13339199 | 1.14 | 0.13 | 0.32340 | -++ | 24.2 |
| rs193695 | 0.94 | 0.06 | 0.32410 | +-- | 0 |
| rs12924331 | 1.06 | 0.06 | 0.32650 | +-+ | 46.6 |
| rs289743 | 1.07 | 0.07 | 0.32800 | +++ | 0 |
| rs34218679 | 1.09 | 0.09 | 0.32910 | -++ | 0 |
| rs247614 | 1.07 | 0.07 | 0.33000 | ++- | 0 |
| rs74931918 | 1.17 | 0.16 | 0.33020 | +++ | 0 |
| rs190324 | 0.94 | 0.06 | 0.33060 | --+ | 0 |
| rs56816073 | 1.07 | 0.07 | 0.33200 | ++- | 0 |
| rs9925265 | 1.06 | 0.06 | 0.33440 | +-+ | 38.7 |
| rs821470 | 0.94 | 0.06 | 0.34020 | --+ | 57.2 |
| rs289718 | 1.06 | 0.07 | 0.34430 | +++ | 0 |
| rs289719 | 1.06 | 0.07 | 0.34430 | +++ | 0 |
| rs72780003 | 1.13 | 0.13 | 0.34750 | -++ | 35.5 |
| rs1167742 | 0.94 | 0.06 | 0.34760 | --+ | 55.5 |
| rs247611 | 1.06 | 0.07 | 0.35030 | ++- | 6.7 |
| rs16965077 | 0.87 | 0.15 | 0.35110 | --- | 0 |
| rs56353889 | 1.06 | 0.07 | 0.35280 | ++- | 6.6 |
| rs173537 | 1.06 | 0.07 | 0.35650 | ++- | 11.2 |
| rs1651666 | 0.94 | 0.06 | 0.36570 | --+ | 49.6 |
| rs193694 | 1.08 | 0.09 | 0.37040 | ++- | 28.2 |
| rs4474668 | 1.06 | 0.07 | 0.37100 | ++- | 10.2 |
| rs866038 | 1.06 | 0.07 | 0.37100 | ++- | 10.2 |
| rs861884 | 1.06 | 0.07 | 0.37480 | ++- | 8.7 |
| rs4783965 | 0.94 | 0.08 | 0.37950 | --- | 0 |
| rs5882 | 1.06 | 0.06 | 0.38030 | +++ | 0 |
| rs117910159 | 0.85 | 0.18 | 0.38260 | +-- | 0 |
| rs12920974 | 0.95 | 0.07 | 0.38690 | --- | 0 |
| rs955513 | 0.95 | 0.06 | 0.38930 | -+- | 16 |
| rs117427818 | 1.14 | 0.15 | 0.39120 | ++- | 39.8 |
| rs75911530 | 1.21 | 0.23 | 0.39520 | ++- | 0 |
| rs34946873 | 1.11 | 0.12 | 0.39740 | +-+ | 55 |
| rs247612 | 1.06 | 0.07 | 0.39750 | ++- | 0 |
| rs1820787 | 1.06 | 0.07 | 0.39760 | ++- | 0 |
| rs736274 | 1.08 | 0.10 | 0.40520 | +++ | 0 |
| rs247613 | 1.06 | 0.07 | 0.40650 | ++- | 0 |
| rs16970107 | 0.94 | 0.07 | 0.41030 | --+ | 48.4 |
| rs2133783 | 1.05 | 0.07 | 0.41850 | ++- | 23.6 |
| rs952440 | 1.05 | 0.07 | 0.41850 | ++- | 23.6 |
| rs37024 | 0.95 | 0.06 | 0.42090 | -+- | 4.7 |
| rs1864163 | 0.95 | 0.07 | 0.42110 | -+- | 0 |
| rs289716 | 1.05 | 0.07 | 0.42210 | +++ | 0 |
| rs16965150 | 0.84 | 0.22 | 0.42370 | 0 | 0 |
| rs2115429 | 0.94 | 0.08 | 0.42840 | -+- | 0 |
| rs37023 | 1.05 | 0.07 | 0.42950 | ++- | 35.2 |
| rs289715 | 1.08 | 0.09 | 0.43000 | +++ | 0 |
| rs12720873 | 1.16 | 0.20 | 0.44100 | ++- | 0 |
| rs16942393 | 0.96 | 0.06 | 0.44590 | +-- | 0 |
| rs8058353 | 1.13 | 0.16 | 0.44950 | +++ | 0 |
| rs8059595 | 1.13 | 0.16 | 0.44950 | +++ | 0 |
| rs36229787 | 0.95 | 0.07 | 0.45140 | --+ | 0 |
| rs28495885 | 0.93 | 0.10 | 0.45220 | -+- | 0 |
| rs7203286 | 0.95 | 0.06 | 0.45290 | --- | 0 |
| rs3764263 | 0.96 | 0.06 | 0.45570 | -+- | 0 |
| rs12720897 | 0.91 | 0.13 | 0.45660 | +-+ | 72.9 |
| rs247606 | 1.07 | 0.08 | 0.45690 | ++- | 57.5 |
| rs193693 | 1.06 | 0.08 | 0.45940 | ++- | 54.3 |
| rs12708983 | 1.17 | 0.22 | 0.46050 | ++- | 0 |
| rs6499863 | 0.94 | 0.08 | 0.46090 | -+- | 0 |
| rs2518058 | 1.06 | 0.08 | 0.46140 | ++- | 59.3 |
| rs2052880 | 0.96 | 0.06 | 0.46370 | +-+ | 0 |
| rs7185561 | 1.05 | 0.07 | 0.46480 | ++- | 0 |
| rs12720898 | 0.91 | 0.13 | 0.46540 | +-+ | 74.7 |
| rs289748 | 1.04 | 0.06 | 0.46790 | -++ | 0 |
| rs711747 | 0.96 | 0.06 | 0.46810 | -+- | 0 |
| rs247609 | 1.05 | 0.07 | 0.46880 | ++- | 24.3 |
| rs12446867 | 1.05 | 0.07 | 0.46950 | ++- | 0 |
| rs17239354 | 0.91 | 0.13 | 0.46960 | +-+ | 72.4 |
| rs12373120 | 0.95 | 0.07 | 0.47300 | --+ | 22 |
| rs9931176 | 1.05 | 0.07 | 0.47440 | ++- | 0 |
| rs58124158 | 0.96 | 0.06 | 0.47870 | --+ | 52.3 |
| rs952439 | 1.06 | 0.08 | 0.48010 | ++- | 56.9 |
| rs821465 | 0.95 | 0.07 | 0.48170 | -+- | 0 |
| rs1820788 | 1.05 | 0.07 | 0.48180 | ++- | 27.5 |
| rs12720918 | 0.96 | 0.07 | 0.48520 | --+ | 0 |
| rs37029 | 0.96 | 0.06 | 0.48530 | -+- | 0 |
| rs37030 | 0.96 | 0.06 | 0.48530 | -+- | 0 |
| rs1428847 | 0.96 | 0.06 | 0.48540 | -+- | 0 |
| rs4784738 | 0.88 | 0.18 | 0.49100 | +-- | 12.4 |
| rs3903056 | 1.06 | 0.08 | 0.49130 | ++- | 54.2 |
| rs8059431 | 1.12 | 0.17 | 0.49370 | ++- | 0 |
| rs1167514 | 0.95 | 0.07 | 0.49730 | +-- | 0 |
| rs247608 | 1.06 | 0.09 | 0.49860 | ++- | 58 |
| rs289707 | 0.95 | 0.07 | 0.50100 | -+- | 0 |
| rs289703 | 1.05 | 0.07 | 0.50320 | ++- | 0 |
| rs16942394 | 1.04 | 0.06 | 0.50340 | +++ | 0 |
| rs881598 | 1.06 | 0.08 | 0.50530 | ++- | 49.8 |
| rs2518056 | 0.96 | 0.06 | 0.50540 | -+- | 0 |
| rs3812963 | 1.06 | 0.09 | 0.51130 | -+- | 10.4 |
| rs62035509 | 0.95 | 0.08 | 0.51430 | -+- | 21.6 |
| rs3794647 | 0.96 | 0.06 | 0.51640 | -+- | 0 |
| rs711748 | 0.96 | 0.06 | 0.51640 | -+- | 0 |
| rs37026 | 0.96 | 0.06 | 0.51740 | -+- | 0 |
| rs289747 | 1.04 | 0.06 | 0.51790 | ++- | 0 |
| rs1801706 | 0.95 | 0.08 | 0.51820 | --+ | 0 |
| rs9924286 | 0.96 | 0.06 | 0.51890 | -+- | 0 |
| rs9926292 | 0.96 | 0.06 | 0.51890 | -+- | 0 |
| rs3794648 | 0.96 | 0.06 | 0.51910 | -+- | 7.3 |
| rs4784749 | 0.95 | 0.08 | 0.52130 | --+ | 27.1 |
| rs2217332 | 1.06 | 0.08 | 0.52310 | ++- | 56.9 |
| rs7196436 | 0.91 | 0.15 | 0.52670 | +-- | 0 |
| rs12598913 | 0.96 | 0.06 | 0.52970 | --+ | 55.5 |
| rs17245715 | 0.94 | 0.10 | 0.53240 | +-- | 9.6 |
| rs1366544 | 1.05 | 0.08 | 0.53380 | ++- | 57.2 |
| rs4548848 | 0.96 | 0.06 | 0.53980 | -+- | 0 |
| rs176532 | 0.96 | 0.07 | 0.54270 | -+- | 13.7 |
| rs75429044 | 0.94 | 0.09 | 0.54520 | -+- | 0 |
| rs2562126 | 1.05 | 0.08 | 0.55100 | ++- | 50.5 |
| rs55664802 | 0.95 | 0.09 | 0.55110 | ++- | 63 |
| rs74023644 | 0.91 | 0.16 | 0.55230 | +-- | 0 |
| rs74023645 | 0.91 | 0.16 | 0.55230 | +-- | 0 |
| rs16965220 | 0.96 | 0.06 | 0.55270 | --+ | 56.5 |
| rs3812964 | 0.97 | 0.06 | 0.55270 | -+- | 0 |
| rs12596509 | 0.97 | 0.06 | 0.55320 | -+- | 0 |
| rs708270 | 0.97 | 0.06 | 0.55320 | -+- | 0 |
| rs711749 | 0.97 | 0.06 | 0.55320 | -+- | 0 |
| rs821466 | 1.04 | 0.06 | 0.55600 | +-- | 19.3 |
| rs2518055 | 1.05 | 0.09 | 0.55830 | ++- | 62.2 |
| rs37031 | 0.97 | 0.06 | 0.55880 | -+- | 0 |
| rs17369468 | 0.96 | 0.07 | 0.56980 | --+ | 72.2 |
| rs62035538 | 0.96 | 0.07 | 0.57000 | --+ | 32.2 |
| rs55634433 | 0.92 | 0.15 | 0.57080 | +-- | 0 |
| rs75974417 | 0.96 | 0.07 | 0.57220 | --+ | 0 |
| rs247607 | 1.05 | 0.08 | 0.57390 | ++- | 62.3 |
| rs5805 | 0.97 | 0.06 | 0.57750 | -+- | 4 |
| rs56079121 | 0.95 | 0.09 | 0.58250 | -+- | 0 |
| rs11076176 | 1.05 | 0.08 | 0.58280 | -++ | 0 |
| rs39718 | 0.97 | 0.06 | 0.58610 | -+- | 0 |
| rs55726180 | 1.03 | 0.06 | 0.59180 | +-+ | 34.5 |
| rs58337780 | 0.92 | 0.16 | 0.59220 | +-- | 0 |
| rs12149572 | 1.03 | 0.06 | 0.59380 | --+ | 15 |
| rs1651665 | 0.97 | 0.07 | 0.59500 | --+ | 65 |
| rs9931252 | 0.95 | 0.09 | 0.59580 | -+- | 0 |
| rs711746 | 0.97 | 0.06 | 0.59610 | -+- | 0 |
| rs9932164 | 0.95 | 0.09 | 0.59750 | -+- | 0 |
| rs289754 | 0.97 | 0.06 | 0.59820 | +-+ | 0 |
| rs9927820 | 1.03 | 0.06 | 0.59860 | +-+ | 27 |
| rs112039804 | 1.06 | 0.11 | 0.59940 | -++ | 0 |
| rs13330423 | 0.97 | 0.06 | 0.60360 | --+ | 0 |
| rs11863728 | 1.03 | 0.06 | 0.60370 | +-+ | 26.3 |
| rs12708968 | 0.95 | 0.10 | 0.60460 | +-- | 4.2 |
| rs12598522 | 1.03 | 0.06 | 0.60610 | -0+ | 6.3 |
| rs718620 | 0.95 | 0.10 | 0.60770 | -+- | 0 |
| rs28168 | 0.97 | 0.06 | 0.60860 | -++ | 2.4 |
| rs17370142 | 0.95 | 0.10 | 0.62360 | -+- | 63.3 |
| rs5808 | 0.97 | 0.06 | 0.62620 | -++ | 6.6 |
| rs13335668 | 0.95 | 0.10 | 0.62800 | ++- | 63.4 |
| rs72780004 | 0.97 | 0.07 | 0.62840 | --+ | 0 |
| rs1167741 | 0.97 | 0.06 | 0.63080 | --+ | 0 |
| rs37027 | 0.97 | 0.06 | 0.63100 | -+- | 25 |
| rs66495554 | 1.03 | 0.07 | 0.63350 | -+- | 0 |
| rs28880001 | 1.03 | 0.06 | 0.63540 | +-+ | 20.7 |
| rs12149414 | 1.03 | 0.06 | 0.63640 | +-+ | 20.6 |
| rs12149520 | 1.03 | 0.06 | 0.63640 | +-+ | 20.6 |
| rs3764262 | 1.03 | 0.06 | 0.63910 | +-+ | 21.4 |
| rs2399594 | 1.03 | 0.06 | 0.64040 | +-+ | 0 |
| rs56172892 | 0.95 | 0.10 | 0.64400 | -+- | 59.1 |
| rs11866974 | 1.03 | 0.06 | 0.64440 | +-+ | 12.7 |
| rs2518054 | 1.04 | 0.09 | 0.64590 | -+- | 7 |
| rs9927174 | 1.03 | 0.06 | 0.64680 | +-+ | 18.9 |
| rs76994065 | 0.94 | 0.14 | 0.65070 | ++- | 30.4 |
| rs56132500 | 1.06 | 0.12 | 0.65240 | -++ | 0 |
| rs9921645 | 1.03 | 0.06 | 0.65590 | +-+ | 18.3 |
| rs13333567 | 1.03 | 0.06 | 0.66610 | +-+ | 11.2 |
| rs62038195 | 1.03 | 0.06 | 0.66610 | +-+ | 11.2 |
| rs6499862 | 0.97 | 0.08 | 0.67060 | -+- | 0 |
| rs58138751 | 1.06 | 0.13 | 0.67380 | -++ | 0 |
| rs112952893 | 1.04 | 0.09 | 0.67450 | -++ | 0 |
| rs4783963 | 0.96 | 0.10 | 0.67460 | -++ | 39.7 |
| rs74023632 | 0.94 | 0.16 | 0.67490 | +-- | 0 |
| rs74611520 | 1.04 | 0.09 | 0.67580 | -+- | 10.9 |
| rs28438857 | 1.04 | 0.10 | 0.67760 | ++- | 50.9 |
| rs34531240 | 1.04 | 0.10 | 0.67760 | ++- | 50.9 |
| rs17369768 | 1.03 | 0.07 | 0.67840 | --+ | 50.2 |
| rs173538 | 0.97 | 0.06 | 0.67850 | -++ | 0 |
| rs6499861 | 0.97 | 0.08 | 0.68360 | -+- | 0 |
| rs60169561 | 0.97 | 0.07 | 0.68840 | --+ | 64 |
| rs72786781 | 0.91 | 0.25 | 0.69420 | -+- | 0 |
| rs117199686 | 0.95 | 0.14 | 0.69470 | ++- | 36.2 |
| rs7198642 | 1.03 | 0.07 | 0.69540 | -+- | 13.1 |
| rs11642606 | 0.98 | 0.06 | 0.69850 | --+ | 0 |
| rs1549670 | 0.98 | 0.06 | 0.69980 | --+ | 22 |
| rs72773119 | 0.94 | 0.15 | 0.70100 | +-- | 0 |
| rs72773120 | 0.94 | 0.15 | 0.70100 | +-- | 0 |
| rs12708967 | 0.97 | 0.08 | 0.70330 | -++ | 0 |
| rs12934632 | 0.97 | 0.08 | 0.70990 | --- | 0 |
| rs8056195 | 1.02 | 0.06 | 0.71110 | +-+ | 25.2 |
| rs9938413 | 1.03 | 0.09 | 0.71190 | -+- | 0 |
| rs72778395 | 1.05 | 0.13 | 0.71880 | -++ | 0 |
| rs11076175 | 1.03 | 0.08 | 0.72010 | -+- | 61.6 |
| rs62035546 | 0.97 | 0.10 | 0.72510 | ++- | 59.6 |
| rs62035547 | 0.97 | 0.10 | 0.72510 | ++- | 59.6 |
| rs9931755 | 1.02 | 0.06 | 0.72880 | +-+ | 0 |
| rs55958623 | 1.03 | 0.09 | 0.73290 | ++- | 68.2 |
| rs12444708 | 1.04 | 0.11 | 0.74060 | -++ | 0 |
| rs37028 | 1.02 | 0.06 | 0.74230 | +-+ | 0 |
| rs28439729 | 0.95 | 0.14 | 0.74340 | ++- | 39 |
| rs9938543 | 0.97 | 0.10 | 0.74640 | ++- | 54.9 |
| rs72773124 | 0.95 | 0.16 | 0.75040 | +-- | 0 |
| rs7195863 | 0.95 | 0.16 | 0.75200 | +-- | 0 |
| rs11076174 | 1.03 | 0.11 | 0.75460 | -++ | 61.2 |
| rs12720922 | 1.02 | 0.08 | 0.75480 | -+- | 62.5 |
| rs176533 | 1.02 | 0.06 | 0.75480 | +-- | 14.1 |
| rs9937834 | 0.97 | 0.10 | 0.75750 | ++- | 57.8 |
| rs56096618 | 1.04 | 0.13 | 0.75850 | -++ | 0 |
| rs34760410 | 0.97 | 0.11 | 0.76310 | +-+ | 20.7 |
| rs289708 | 0.98 | 0.08 | 0.76560 | -++ | 0 |
| rs291040 | 0.98 | 0.06 | 0.76990 | +-+ | 0 |
| rs9923854 | 0.97 | 0.11 | 0.77100 | -++ | 33.9 |
| rs7499892 | 1.02 | 0.08 | 0.77580 | -+- | 65.5 |
| rs1991515 | 0.98 | 0.06 | 0.79010 | -++ | 27.7 |
| rs9930761 | 0.97 | 0.13 | 0.79220 | -++ | 33 |
| rs11644125 | 0.98 | 0.06 | 0.79850 | --+ | 65.6 |
| rs12445769 | 1.02 | 0.06 | 0.79860 | +-+ | 11.5 |
| rs7499911 | 0.96 | 0.14 | 0.80040 | ++- | 43.8 |
| rs5883 | 0.97 | 0.15 | 0.81220 | -++ | 60.1 |
| rs12149408 | 1.02 | 0.06 | 0.81240 | +-+ | 0 |
| rs28888131 | 0.98 | 0.08 | 0.81300 | -+- | 0 |
| rs9788873 | 0.99 | 0.06 | 0.81520 | +-+ | 16.7 |
| rs12928552 | 1.03 | 0.12 | 0.81890 | -++ | 0 |
| rs1566439 | 1.01 | 0.06 | 0.81980 | --+ | 0 |
| rs7204290 | 0.99 | 0.06 | 0.82070 | +-+ | 25.2 |
| rs821463 | 1.01 | 0.06 | 0.82460 | +-- | 6.3 |
| rs56315364 | 1.01 | 0.06 | 0.82880 | --+ | 26.8 |
| rs72771478 | 0.95 | 0.23 | 0.83030 | -+- | 0 |
| rs72771479 | 0.95 | 0.23 | 0.83030 | -+- | 0 |
| rs12708974 | 1.02 | 0.10 | 0.83470 | +-+ | 55.5 |
| rs62035542 | 0.98 | 0.10 | 0.83840 | ++- | 65.5 |
| rs2399597 | 0.98 | 0.09 | 0.84060 | -++ | 0 |
| rs74439742 | 0.98 | 0.08 | 0.84680 | --+ | 9.4 |
| rs117426126 | 0.97 | 0.15 | 0.84760 | ++- | 39.3 |
| rs76691037 | 0.97 | 0.16 | 0.84890 | +-- | 0 |
| rs863706 | 1.01 | 0.07 | 0.85560 | ++- | 35.9 |
| rs80195568 | 1.03 | 0.14 | 0.85600 | ++- | 0 |
| rs75378421 | 0.97 | 0.14 | 0.85750 | ++- | 28.1 |
| rs118146573 | 1.02 | 0.09 | 0.85860 | -+- | 0 |
| rs1651667 | 0.99 | 0.07 | 0.85910 | --+ | 66.4 |
| rs80327887 | 0.98 | 0.14 | 0.85920 | ++- | 0 |
| rs78459786 | 0.98 | 0.14 | 0.86240 | ++- | 37.2 |
| rs16965070 | 0.98 | 0.12 | 0.86280 | -++ | 0 |
| rs9929488 | 0.99 | 0.07 | 0.86460 | -+- | 60.2 |
| rs56285233 | 0.98 | 0.12 | 0.86890 | -++ | 0 |
| rs16965037 | 1.01 | 0.06 | 0.87340 | --+ | 58.5 |
| rs62038194 | 1.01 | 0.07 | 0.88320 | ++- | 31.6 |
| rs16965039 | 1.02 | 0.12 | 0.88380 | -+- | 0 |
| rs62035543 | 0.99 | 0.10 | 0.88690 | ++- | 65.4 |
| rs4544228 | 1.01 | 0.07 | 0.88820 | ++- | 40.5 |
| rs116889966 | 1.02 | 0.14 | 0.89070 | ++- | 0 |
| rs1151265 | 1.02 | 0.13 | 0.89260 | -+- | 0 |
| rs16965033 | 0.98 | 0.12 | 0.89270 | -+- | 0 |
| rs80103996 | 1.02 | 0.14 | 0.89800 | ++- | 0 |
| rs36229786 | 0.99 | 0.08 | 0.90440 | -++ | 0 |
| rs17310296 | 0.98 | 0.14 | 0.90770 | ++- | 34.2 |
| rs8044804 | 0.99 | 0.06 | 0.91290 | +-+ | 25.9 |
| rs11861555 | 1.01 | 0.06 | 0.91590 | +-+ | 0 |
| rs62035537 | 0.99 | 0.09 | 0.91610 | -++ | 3.3 |
| rs62035545 | 1.01 | 0.10 | 0.92080 | ++- | 65.8 |
| rs7500979 | 0.99 | 0.06 | 0.92890 | +-+ | 33.7 |
| rs7205692 | 0.99 | 0.09 | 0.93500 | -++ | 0 |
| rs9939318 | 0.99 | 0.10 | 0.94140 | ++- | 63.1 |
| rs11864751 | 1.00 | 0.06 | 0.96150 | +-+ | 28.6 |
| rs74912812 | 0.99 | 0.14 | 0.96220 | ++- | 46.4 |
| rs289737 | 1.00 | 0.07 | 0.96920 | ++- | 0 |
| rs1167513 | 1.00 | 0.13 | 0.97280 | -+- | 0 |
| rs291042 | 1.00 | 0.08 | 0.97420 | -+- | 0 |
| rs62035544 | 1.00 | 0.10 | 0.97520 | ++- | 61.3 |
| rs76631084 | 1.00 | 0.14 | 0.97580 | ++- | 0 |
| rs17369578 | 1.00 | 0.14 | 0.97700 | ++- | 57.9 |
| rs289752 | 1.00 | 0.06 | 0.97820 | +-- | 39.6 |
| rs74613568 | 1.00 | 0.14 | 0.98330 | ++- | 0 |
| rs55744249 | 1.00 | 0.09 | 0.98600 | ++- | 72.4 |
| rs56273021 | 1.00 | 0.06 | 0.98790 | --+ | 62.8 |
| OR = odds ratio, SE = standard error, SNP = single nucleotide polymorphism, + = variant increases ICH risk, - = variant decreases ICH risk. | | | | | |

| **Supplementary Table S5. Discovery phase results for top SNPs within each semi-independent *CETP* locus with ICH risk** | | | | | | | | | | |
| --- | --- | --- | --- | --- | --- | --- | --- | --- | --- | --- |
| **Allele information** | | | | **Meta-analysis results** | | | | **Study specific odds ratios** | | |
| **Lead SNP** | **CHR** | **BPP** | **Tested allele** | **OR** | **SE** | **P** | **I^2^** | **GOCHA** | **ISGC ICH** | **GERFHS** |
| rs173539 | 16 | 56988044 | T | 1.25 | 0.0646 | 0.00060 | 0 | 1.19 | 1.34 | 1.22 |
| rs820299 | 16 | 57000284 | G | 0.81 | 0.0628 | 0.00075 | 48 | 0.82 | 0.71 | 0.97 |
| rs158478 | 16 | 57007734 | A | 1.21 | 0.0611 | 0.00148 | 56 | 1.16 | 1.43 | 1.05 |
| rs4784745 | 16 | 57014875 | G | 0.85 | 0.0635 | 0.00973 | 0 | 0.89 | 0.80 | 0.87 |
| rs7187261 | 16 | 57031716 | T | 1.46 | 0.1588 | 0.01636 | 0 | 1.34 | 1.67 | 1.33 |
| rs289751 | 16 | 57026775 | G | 1.50 | 0.1777 | 0.02310 | 19 | 1.48 | 1.93 | 0.96 |
| rs711751 | 16 | 56993909 | A | 0.87 | 0.0617 | 0.02725 | 0 | 0.88 | 0.83 | 0.93 |
| rs4783962 | 16 | 56995038 | T | 0.86 | 0.0697 | 0.02749 | 0 | 0.89 | 0.80 | 0.91 |
| rs891144 | 16 | 57011936 | T | 1.81 | 0.2872 | 0.03975 | 0 | 1.77 | 2.09 | 1.48 |
| rs71387147 | 16 | 57010382 | G | 0.77 | 0.1269 | 0.04162 | 0 | 0.79 | 0.77 | 0.75 |
| rs1800775 | 16 | 56995236 | A | 1.12 | 0.058 | 0.04412 | 0 | 1.20 | 1.11 | 1.05 |
| rs289746 | 16 | 57020205 | T | 1.16 | 0.0728 | 0.04637 | 30 | 1.38 | 1.07 | 1.05 |
| Association results by locus for variants displaying association with ICH with p<0.05, clumped into regions with r^2^>0.5. BPP = base pair position, CHR = chromosome, GERFHS = Genetic and Environmental Risk Factors for Hemorrhagic Stroke study, GOCHA = Genes and Outcomes of Cerebral Hemorrhage on Anticoagulation study, ISGC = International Stroke Genetics Consortium, OR = odds ratio, SE = standard error, SNP = single nucleotide polymorphism | | | | | | | | | | |

| **Supplementary Table S6. Discovery phase association results for top SNPs within each semi-independent *CETP* locus, stratified by ICH location** | | | | | | | | | | | | | | |
| --- | --- | --- | --- | --- | --- | --- | --- | --- | --- | --- | --- | --- | --- | --- |
| **Allele information** | | | **All ICH** | | | | **Lobar ICH** | | | | **Non-lobar ICH** | | | |
| **SNP** | **CHR** | **Tested  allele** | **OR** | **SE** | **p** | **I^2^** | **OR** | **SE** | **p** | **I^2^** | **OR** | **SE** | **p** | **I^2^** |
| rs173539 | 16 | T | 1.25 | 0.06 | 0.00060 | 0 | 1.27 | 0.08 | 0.00309 | 0 | 1.22 | 0.08 | 0.01151 | 0 |
| rs820299 | 16 | G | 0.81 | 0.06 | 0.00075 | 48 | 0.83 | 0.08 | 0.02348 | 0 | 0.80 | 0.08 | 0.00348 | 55 |
| rs158478 | 16 | A | 1.21 | 0.06 | 0.00148 | 56 | 1.12 | 0.08 | 0.12610 | 0 | 1.28 | 0.07 | 0.00067 | 70 |
| rs4784745 | 16 | G | 0.85 | 0.06 | 0.00973 | 0 | 0.81 | 0.08 | 0.01099 | 0 | 0.86 | 0.08 | 0.05479 | 0 |
| rs7187261 | 16 | T | 1.46 | 0.16 | 0.01636 | 0 | 1.49 | 0.20 | 0.04468 | 0 | 1.40 | 0.19 | 0.08000 | 17 |
| rs289751 | 16 | G | 1.50 | 0.18 | 0.02310 | 19 | 1.16 | 0.24 | 0.53900 | 0 | 1.71 | 0.20 | 0.00761 | 45 |
| rs711751 | 16 | A | 0.87 | 0.06 | 0.02725 | 0 | 0.83 | 0.08 | 0.02132 | 0 | 0.92 | 0.07 | 0.27480 | 0 |
| rs4783962 | 16 | T | 0.86 | 0.07 | 0.02749 | 0 | 0.88 | 0.09 | 0.16880 | 0 | 0.83 | 0.08 | 0.02928 | 8 |
| rs891144 | 16 | T | 1.81 | 0.29 | 0.03975 | 0 | 2.02 | 0.53 | 0.18860 | 0 | 2.45 | 0.49 | 0.06926 | 0 |
| rs71387147 | 16 | G | 0.77 | 0.13 | 0.04162 | 0 | 0.75 | 0.17 | 0.08241 | 0 | 0.81 | 0.15 | 0.16250 | 0 |
| rs1800775 | 16 | A | 1.12 | 0.06 | 0.04412 | 0 | 1.14 | 0.07 | 0.06925 | 0 | 1.13 | 0.07 | 0.08727 | 0 |
| rs289746 | 16 | T | 1.16 | 0.07 | 0.04637 | 30 | 1.17 | 0.09 | 0.08431 | 60 | 1.15 | 0.09 | 0.09409 | 0 |
| CHR = chromosome, OR = odds ratio, SE = standard error, SNP = single nucleotide polymorphism | | | | | | | | | | | | | | |

| Supplementary Table S7. International Stroke Genetics Consortium Contributors | |
| --- | --- |
| Name | **Affiliation and ISGC Role** |
| Sylvia Smoller, PhD | Albert Einstein College of Medicine, Site co-investigator |
| John Sorkin, MD | Baltimore VA Medical Center, Site co-investigator |
| Xingwu Wang, MD | Beijing Hypertension League Institute, Site co-investigator |
| Magdy Selim, MD, PhD | Beth Israel Deaconess Medical Center, Site co-investigator |
| Aleksandra Pikula, MD, PhD | Boston University Medical Center, Site co-investigator |
| Philip Wolf, MD, PhD | Boston University School of Medicine, Site co-investigator |
| Stephanie Debette, MD | Boston University School of Medicine, Site co-investigator |
| Sudha Seshadri, MD | Boston University School of Medicine, Site co-investigator |
| Paul de Bakker, PhD | Brigham and Women's Hospital, Site co-investigator |
| Daniel Chasman, MD | Brigham and Women's Hospital, Site co-investigator |
| Kathryn Rexrode, MD | Brigham and Women's Hospital, Harvard Medical School, Site co-investigator |
| Ida Chen, MD | Cedars Sinai Medical Center, Site co-investigator |
| Jerome Rotter, MD | Cedars Sinai Medical Center, Site co-investigator |
| May Luke, MD | Celera, Site co-investigator |
| Michelle Sale, MD | University of Virginia, Site co-investigator |
| Tsong-Hai Lee, MD | Chang Gung Memorial Hospital, Linkou Medical Center, Site co-investigator |
| Ku-Chou Chang, MD | Chang Gung Memorial Hospital, Chang Gung University, Site co-investigator |
| Mitchell Elkind, MD, MS | Columbia University, Site co-investigator |
| Larry Goldstein, MD, PhD | Duke University, Site co-investigator |
| Michael Luke James, MD | Duke University, Site co-investigator |
| Monique Breteler, MD | Erasmus University, Site co-investigator |
| Chris O'Donnell, MD | Framingham Heart Study, Site co-investigator |
| Didier Leys, MD | France, Site co-investigator |
| Cara Carty, MD | Fred Hutchinson Cancer Research Center, Site co-investigator |
| Chelsea Kidwell, MD | Georgetown University, Site co-investigator |
| Jes Olesen, MD | Glostrup Hospital, Site co-investigator |
| Pankaj Sharma, MD, PhD | Hammersmith Hospitals & Imperial College London, Site co-investigator |
| Stephen Rich, MD, PhD | University of Virginia Health System, Site co-investigator |
| Turgot Tatlisumak, MD | Helsinki University Central Hospital, Site co-investigator |
| Olli Happola, MD | Helsinki University Central Hospital, Site co-investigator |
| Philippe Bijlenga, MD | Hìpitaux Universityersitaires de Genäve, Site co-investigator |
| Carolina Soriano, MD | IMIM-Hospital del Mar, Site co-investigator |
| Eva Giralt, MD | IMIM-Hospital del Mar, Site co-investigator |
| Jaume Roquer, MD | IMIM-Hospital del Mar , Site co-investigator |
| Jordi Jimenez-Conde, MD | IMIM-Hospital del Mar , Site co-investigator |
| Ioana Cotlarcius, MD | Imperial College London, Site co-investigator |
| John Hardy, MD | Institute of Neurology, UCL, Site co-investigator |
| Michal Korostynski, MD | Institute of Pharmacology, Krakow, Poland , Site co-investigator |
| Giorgio Boncoraglio, MD | IRCCS Istituto neurologico Carlo Besta , Site co-investigator |
| Elena Ballabio, MD | IRCCS Istituto neurologico Carlo Besta , Site co-investigator |
| Eugenio Parati, MD | IRCCS Istituto neurologico Carlo Besta , Site co-investigator |
| Adamski Mateusz, MD | Jagiellonian University, Site co-investigator |
| Andrzej Urbanik, MD | Jagiellonian University, Site co-investigator |
| Tomasz Dziedzic, MD | Jagiellonian University, Site co-investigator |
| Jeremiasz Jagiella, MD | Jagiellonian University, Site co-investigator |
| Jerzy Gasowski, MD | Jagiellonian University, Site co-investigator |
| Marcin Wnuk, MD | Jagiellonian University, Site co-investigator |
| Rafael Olszanecki, MD | Jagiellonian University, Site co-investigator |
| Joanna Pera, MD | Jagiellonian University, Site co-investigator |
| Agnieszka Slowik, MD | Jagiellonian University, Site co-investigator |
| Karol Jozef Juchniewicz , MD | Jagiellonian University, Site co-investigator |
| Christopher Levi, MD | John Hunter Hospital, University of Newcastle, Site co-investigator |
| Paul Nyquist, MD, PhD | Johns Hopkins School of Medicine, Scientific committee |
| Iscia Cendes, MD | Joinville Biobank, Site co-investigator |
| Norberto Cabral, MD | Joinville Biobank, Site co-investigator |
| Paulo Franca, MD | Joinville Biobank, Site co-investigator |
| Anderson Goncalves, MD | Joinville Biobank, Site co-investigator |
| Lina Keller, MD | Karolinska Institutet , Site co-investigator |
| Milita Crisby, MD | Karolinska Institutet, Sweden, Site co-investigator |
| Konstantinos Kostulas, MD | Karolinska Institutet, Karolinska University Hospital, Huddinge unit, Site co-investigator |
| Robin Lemmens, MD | Leuven, Site co-investigator |
| Kourosh Ahmadi, MD | London, Site co-investigator |
| Christian Opherk, MD | Ludwig-Maximilians-Univeritat Munchen , Site co-investigator |
| Marco Duering, MD | Ludwig-Maximilians-Univeritat Munchen , Site co-investigator |
| Martin Dichgans, MD | Ludwig-Maximilians-Univeritat Munchen , Site co-investigator |
| Rainer Malik, PhD | Ludwig-Maximilians-Univeritat Munchen , Site co-investigator |
| Mariya Gonik, MD | Ludwig-Maximilians-Univeritat Munchen , Site co-investigator |
| Julie Staals, MD | Maastricht University Medical Centre, Maastricht, the Netherlands, Site co-investigator |
| Olle Melander, MD, PhD | Malmo University Hospital, Site co-investigator |
| Philippe Burri, MD | Malmo University Hospital, Site co-investigator |
| Ariane Sadr-Nabavi, MD | Mashhad University of Medical Sciences, Site co-investigator |
| Javier Romero, MD, PhD | Massachusetts General Hospital, Site co-investigator |
| Alessandro Biffi, MD | Massachusetts General Hospital, Site co-investigator |
| Chris Anderson, MD | Massachusetts General Hospital, Site co-investigator |
| Guido Falcone, MD | Massachusetts General Hospital, Site co-investigator |
| Bart Brouwers, MD | Massachusetts General Hospital, Site co-investigator |
| Jonathan Rosand, MD, MSc | Massachusetts General Hospital, Site co-investigator |
| Natalia Rost, MD, MSc | Massachusetts General Hospital, Site co-investigator |
| Rose Du, MD | Massachusetts General Hospital, Site co-investigator |
| Christina Kourkoulis, BA | Massachusetts General Hospital, Site co-investigator |
| Thomas Battey, BA | Massachusetts General Hospital, Site co-investigator |
| Steven Lubitz, MD, PhD | Massachusetts General Hospital, Site co-investigator |
| Bertram Mueller-Myhsok, MD | Max Planck Institute of Psychiatry, Munich, Site co-investigator |
| James Meschia, MD | Mayo Clinic, Steering committee |
| Thomas Brott, MD, PhD | Mayo Clinic, Site co-investigator |
| Guillaume Pare, MD | McMaster University, Steering committee, Scientific committee |
| Alexander Pichler, MD | Medical University Graz, Site co-investigator |
| Christian Enzinger, MD | Medical University Graz, Site co-investigator |
| Helena Schmidt, MD | Medical University Graz, Site co-investigator |
| Reinhold Schmidt, MD | Medical University Graz, Site co-investigator |
| Stephan Seiler, MD | Medical University Graz, Site co-investigator |
| Susan Blanton, MD | Miami Institute of Human Genomics, University of Miami Miller School of Medicine, Site co-investigator |
| Yoshiji Yamada, MD | Mie University, Site co-investigator |
| Anna Bersano, MD | Milan University, Site co-investigator |
| Tatjana Rundek, MD | University of Miami, Site co-investigator |
| Ralph Sacco, MD | University of Miami, Site co-investigator |
| Yu-Feng Yvonne Chan, MD | Mount Sinai Medical Center, Site co-investigator |
| Andreas Gschwendtner, MD, PhD | Ludwig-Maximilians-Univeritat Munchen, Site co-investigator |
| Zhen Deng, MD | Nanfang Hospital, Southern Medical University, Site co-investigator |
| Taura Barr, MD | National Institutes of Health, Site co-investigator |
| Katrina Gwinn, MD | National Institutes of Health, Site co-investigator |
| Roderick Corriveau, MD | National Institutes of Health, Site co-investigator |
| Andrew Singleton, MD, PhD | National Institutes of Health, Site co-investigator |
| Salina Waddy, MD | National Institutes of Health, Site co-investigator |
| Lenore Launer, MD | National Institutes of Health, Site co-investigator |
| Christopher Chen, MD | National Neuroscience Institute, Singapore General Hospitalˇ, Site co-investigator |
| Kim En Le, MD | National Neuroscience Institute, Singapore General Hospitalˇ, Site co-investigator |
| Wei Ling Lee, MD | National Neuroscience Institute, Singapore General Hospitalˇ, Site co-investigator |
| Eng King Tan, MD | National Neuroscience Institute, Singapore General Hospitalˇ, Site co-investigator |
| Akintomi Olugbodi, MD | Obafemi Awolowo University, Site co-investigator |
| Peter Rothwell, MD, PhD | Oxford, Radcliffe Infirmary, Site co-investigator |
| Sabrina Schilling, MD | Paris, France, Site co-investigator |
| Vincent Mok, MD | Prince of Wales Hospital, The Chinese University of Hong Kong, Site co-investigator |
| Elena Lebedeva, MD | Russia, Site co-investigator |
| Christina Jern, MD | Sahlgrenska University Hospital, Scientific committee |
| Katarina Jood, MD | Sahlgrenska University Hospital, Site co-investigator |
| Sandra Olsson, MD | Sahlgrenska University Hospital, Site co-investigator |
| Helen Kim, MD | San Francisco General Hospital, Center for Cerebrovascular Research, Site co-investigator |
| Chaeyoung Lee, MD | Soongsil University, Site co-investigator |
| Laura Kilarski, MD | St. George's University of London, Site co-investigator |
| Hugh Markus, MD | St. George's, University of London, Site co-investigator |
| Jennifer Peycke, MD | St. George's, University of London, Site co-investigator |
| Steve Bevan, PhD | St. George's, University of London, Site co-investigator |
| Wayne Sheu, MD | Taichung Veterans General Hospital, Site co-investigator |
| Hung Yi Chiou, MD | Taipei Medical University, Site co-investigator |
| Joseph Chern, MD | Taipei Medical University, Site co-investigator |
| Elias Giraldo, MD | The University of Tennessee Health Science Center at Memphis, Site co-investigator |
| Muhammad Taqi, MD | The University of Tennessee Health Science Center at Memphis, Site co-investigator |
| Vivek Jain, MD | UC Irvine Medical Center, Site co-investigator |
| Olivia Lam, MD | University of California San Francisco, Site co-investigator |
| George Howard, MD | University of Alabama School of Public Health, Site co-investigator |
| Daniel Woo, MD | University of Cincinnati, Steering committee |
| Steven Kittner, MD | University of Maryland Hospital, Site co-investigator |
| Braxton Mitchell, PhD, MPH | University of Maryland School of Medicine, Site co-investigator |
| John Cole, MD | University of Maryland School of Medicine, Site co-investigator |
| Jeff O'Connell, MD | University of Maryland School of Medicine, Site co-investigator |
| Dianna Milewicz, MD | University of Texas Medical School at Houston, Site co-investigator |
| Kachikwu Illoh, MD | University of Texas-Houston, Site co-investigator |
| Bradford Worrall, MD | University of Virginia Health System, Site co-investigator |
| Colin Stine, MD | University. of MD School of Medicine, Site co-investigator |
| Bartosz Karaszewski, MD | University College London, Site co-investigator |
| David Werring, MD | University College London, Site co-investigator |
| Reecha Sofat, MD | University College London, Site co-investigator |
| June Smalley, MD | University College London, Site co-investigator |
| Arne Lindgren, MD | University Hospital Lund, Steering committee, Scientific committee |
| Bjorn Hansen, BA | University Hospital Lund, Site co-investigator |
| Bo Norrving, MD | University Hospital Lund, Site co-investigator |
| Gustav Smith, MD | University Hospital Lund, Site co-investigator |
| Juan Jose Martin, MD | University Hospital Sanatorio Allende, Cordoba, Argentine, Site co-investigator |
| Vincent Thijs, MD | University Hospitals Leuven, Site co-investigator |
| Karin Klijn, MD | University Medical Center Utrecht, Site co-investigator |
| Femke van't Hof, MD, PhD | University Medical Center Utrecht, Site co-investigator |
| Ale Algra, MD | University Medical Center Utrecht, Site co-investigator |
| Mary Macleod, MD | University of Aberdeen, Site co-investigator |
| Rodney Perry, MD | University of Alabama at Birmingham School of Public Health, Site co-investigator |
| Donna Arnett, MD | University of Alabama at Birmingham School of Public Health, Site co-investigator |
| Alessandro Pezzini, MD | University of Brescia, Site co-investigator |
| Alessandro Padovani, MD | University of Brescia, Site co-investigator |
| Steve Cramer, MD, PhD | University of California Irvine, Site co-investigator |
| Mark Fisher, MD | University of California Irvine, Site co-investigator |
| Danish Saleheen, MD | University of Pennsylvania, Site co-investigator |
| Joseph Broderick, MD | University of Cincinnati, Site co-investigator |
| Brett Kissela, MD | University of Cincinnati, Site co-investigator |
| Alex Doney, MD | University of Dundee, Site co-investigator |
| Cathie Sudlow, MD | University of Edinburgh, Western General Hospital, Steering committee |
| Kristiina Rannikmae, MD | University of Edinburgh, Western General Hospital, Site co-investigator |
| Scott Silliman, MD | University of Florida, Site co-investigator |
| Caitrin McDonough, MD | University of Florida, Site co-investigator |
| Matthew Walters, MD | University of Glasgow, Site co-investigator |
| Annie Pedersen, MD | University of Gothenburg, Site co-investigator |
| Kazuma Nakagawa, MD | University of Hawaii, Site co-investigator |
| Christy Chang, MD | University of Maryland, Site co-investigator |
| Mark Dobbins, MD | University of Maryland,, Site co-investigator |
| Patrick McArdle, PhD | University of Maryland, Site co-investigator |
| Yu-Ching Chang, MD | University of Maryland, Site co-investigator |
| Robert Brown, MD | University of Michigan, Site co-investigator |
| Devin Brown, MD | University of Michigan, Site co-investigator |
| Elizabeth Holliday, MD | University of Newcastle, Site co-investigator |
| Raj Kalaria, MD | University of Newcastle, Site co-investigator |
| Jane Maguire, MD | University of Newcastle, John Hunter Hospital, Steering committee |
| John Attia, MD | University of Newcastle, John Hunter Hospital, Site co-investigator |
| Martin Farrall, MD | University of Oxford, Wellcome Trust Center for Human Genetics, Site co-investigator |
| Anne-Katrin Giese, MD | University of Rostock, Germany, Site co-investigator |
| Myriam Fornage, MD | University of Texas- Houston, Health Sciences Center, Scientific committee |
| Jennifer Majersik, MD | University of Utah, Scientific committee |
| Mary Cushman, MD | University of Vermont and Fletcher Allen Health Care, Site co-investigator |
| Keith Keene, MD | University of Virginia, USA, Site co-investigator |
| Siiri Bennett, MD | University of Washington, Site co-investigator |
| David Tirschwell, MD, MSc | University of Washington, Site co-investigator |
| Bruce Psaty, MD | University of Washington, USA, Site co-investigator |
| Alex Reiner, MD | University of Washington, USA, Site co-investigator |
| Will Longstreth, MD | University of Washington, Harborview Medical Center, Site co-investigator |
| David Spence, MD | University of Western Ontario, Robarts Research Institute, Site co-investigator |
| Joan Montaner, MD | Vall d?Hebron Hospital, Site co-investigator |
| Israel Fernandez-Cadenas, MD | Vall d?Hebron Hospital, Steering committee |
| Carl Langefeld, MD | Wake Forest University, Site co-investigator |
| Cheryl Bushnell, MD | Wake Forest University Health Sciences, Site co-investigator |
| Laura Heitsch, MD | Washington University of St. Louis, Site co-investigator |
| Jin-Moo Lee, MD, PhD | Washington University of St. Louis, Site co-investigator |
| Kevin Sheth, MD | Yale New Haven Hospital, Yale School of Medicine, Site co-investigator |
